# Synthesis of tunable porosity of fluorine-enriched porous organic polymer materials with excellent CO_2_, CH_4_ and iodine adsorption

**DOI:** 10.1038/s41598-017-14598-0

**Published:** 2017-10-25

**Authors:** Guoyan Li, Chan Yao, Jiku Wang, Yanhong Xu

**Affiliations:** 1 Key Laboratory of Preparation and Applications of Environmental Friendly Materials of the Ministry of Education, Jilin Normal University, Changchun, 130103 China; 2 Key Laboratory of Functional Materials Physics and Chemistry of the Ministry of Education, Jilin Normal University, Siping, 136000 China

## Abstract

We herein report the construction of four the novel fluorine-enriched conjugated microporous polymers (FCMP-600@1-4), which have permanent porous structures and plenty of fluorine atoms in the skeletons as effective sorption sites. Among them, FCMP-600@4 shows considerable adsorption capacity of CO_2_ of 5.35 mmol g^−1^ at 273 K, and 4.18 mmol g^−1^ at 298 K, which is higher than the reported values for most porous polymers. In addition, FCMP-600@1-4 display high selectivity of CO_2_/N_2_ and high CH_4_ uptakes.

## Introduction

Today, world climate change and environmental problems have become increasingly prominent, so that people have to face the impact of excessive carbon dioxide of atmosphere on humanity, such as the global warming and acid rain. People are eager to find a solution to reduce the concentration of carbon dioxide in the atmosphere, while limiting its emissions, but also studying the ability to capture and storage of new materials. For this purpose, porous organic polymers (POPs) are emerged as the times required, which is a new kind of porous materials with large specific surface area and permanent pore structure. Because of its low density, large specific surface area, adjustable size, and high porosity, as well as a great potential in gas storage, separation, heterogeneous catalysis and other aspects^1,2^. POPs has become one of the hotspots in the recent years and rapid development. People have studied a series of POPs, in addition to traditional zeolites^3^ and activated carbons^4^, including polymers of intrinsic microporosity (PIMs)^5^, hypercross-linked polymers (HCPs)^6^, conjugated microporous polymers (CMPs)^7^, and covalent organic frameworks (COFs)^8^. Compared with inorganic microporous materials and metal organic frameworks (MOFs), the synthesis of POPs has just started. But the organic synthesis of chemistry and polymer chemistry have been provided a wide range of development space for the synthesis of such materials. Therefore, from scientific research and practical application, design and synthesis of POPs with good adsorption property of carbon dioxide are of great significance.

Among them, CMPs have attracted a high degree of concern in the recent years due to the excellent capture performance of CMPs for carbon dioxide^1,7–13^. CMPs are synthesized via metal-catalyzed cross-coupling chemistry to form cross-linked network. It is a subclass of POPs with conjugated structure, precise adjustment of micropore, large specific surface area and high stability, and the introduction of functional groups in the pore skeleton can effectively improve the capture capacity of carbon dioxide.

**Figure 1:**
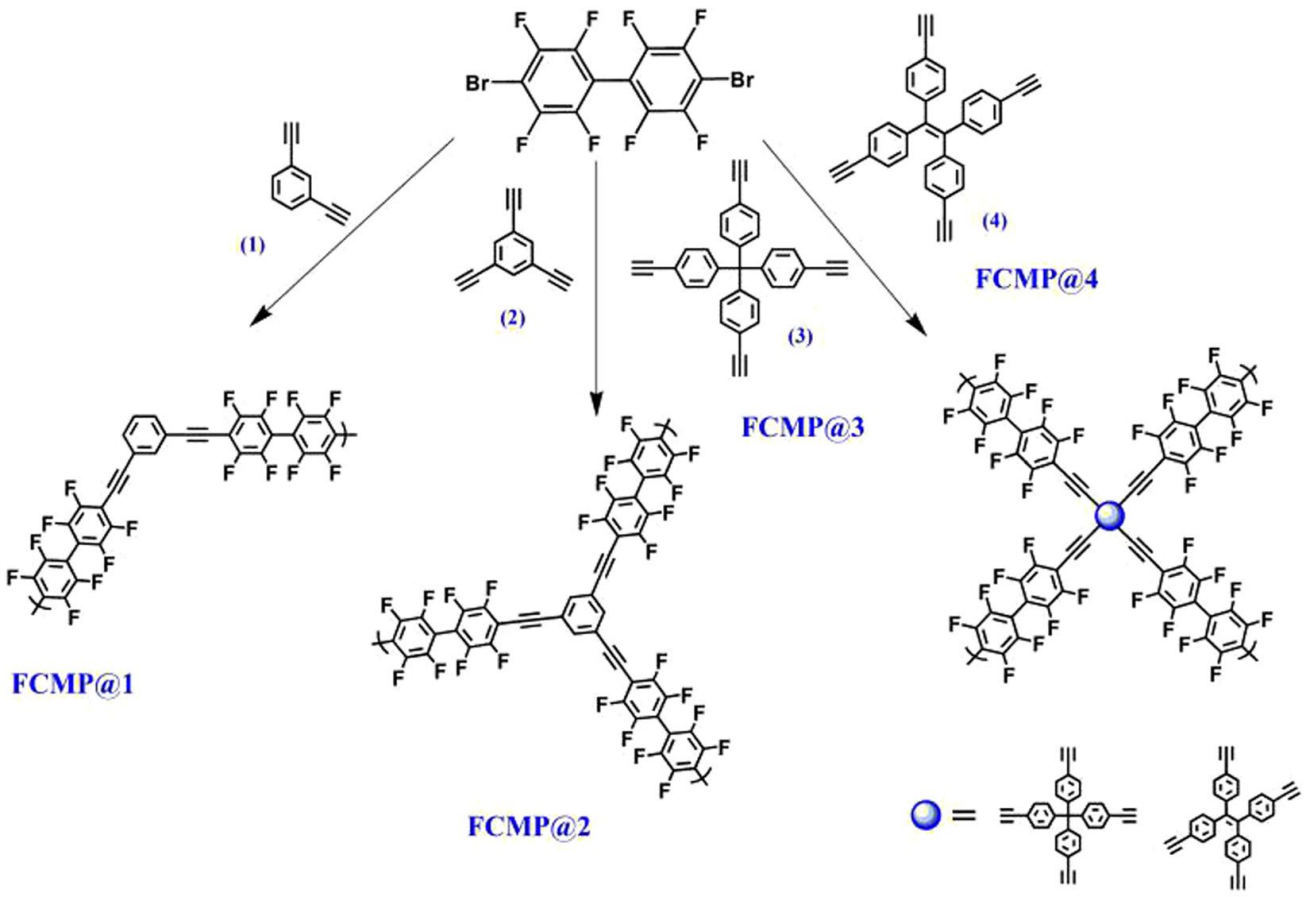
The synthetic routes of FCMP@1-4 polymers.

In particular, the existence of nitrogen atoms in the porous skeleton, the aromatic heterocyclic network, the introduction of ions and so on are all beneficial to improving the adsorption of carbon dioxide on the materials in reported research studies^9–12^. In order to improve the adsorption properties of carbon dioxide on the polymers, in this paper, fluorine-enriched monomer 4,4′-dibromooctafluorobiphenyl (DBFB) and comonomer containing acetylene bond were selected to synthesize a series of structural tunable CMPs by Sonogashira-higihara reaction under Pd(0) catalysis (Fig. 1, FCMP@1-4). Subsequent thermal treatment of these FCMPs precursors at 600 °C, yielded four fluorine-doped porous carbons, and then these materials were denoted FCMP-600@1-4, respectively. The samples not only have abundant fluorine atoms in the skeleton but also can be controlled the pore size and other parameters by adjusting the geometry of comonomers, and then effect the adsorption of CO_2_ of porous materials. For example, by changing the size and the geometry of comonomer with acetylene bond, the BET surface areas of the polymers increase from 755 to 901 m^2^ g^−1^, and the total pore volumes of the polymers hole also vary from 0.4242 to 0.6654 cm^3^ g^−1^. Particularly, FCMP-600@1-4 exhibit the outstanding adsorption of carbon dioxide and methane.

## Results

### Synthesis and characterization

All of the polymer networks were synthesized by palladium(0)-catalyzed Sonogashira-higihara reaction of 4,4′-dibromooctafluorobiphenyl (DBFB) and comonomers containing acetylene moities. All the reactions were carried out at a fixed reaction temperature and reaction time (120 °C/48 h). The general synthetic routes toward FCMP@1-4 polymers are shown in Fig. 1. The insoluble polymers were filtered and washed with water, tetrahydrofunan, chloroform, and methanol, respectively, in order to remove the inorganic salts, organic monomers, residual catalyst, and oligomers. Then the pyrolysis reactions of the FCMP@1-4 were carried out on quartz tubes in an electric furnace under a argon atmosphere. The FCMP@1, 2, 3, and 4 samples were heated from the room temperature to 400 °C, 600 °C, and 800 °C with a heating rate of 3 °C/min, then pyrolyzed at 400 °C, 600 °C, and 800 °C for 2 h in argon gas (400 sccm), respectively. Then, we investigated the CO_2_ adsorption capacity of these samples at 273 K and 298 K, respectively. We found these precursors at 600 °C displayed the best results compared to precursors at 400 °C and 800 °C. Therefore, we selected samples processed under 600 °C condition to be carefully investigated. The pyrolysis reactions at 600 °C in argon gas were denoted to FCMP-600@1, FCMP-600@2, FCMP-600@3, and FCMP-600@4. Our aim is to explore the effect of structure and connecting position of linker on pore properties of the resulting porous polymers. All of these polymers are insoluble in common organic solvents because of their highly cross linked structures.

Formation of FCMP@1-4 was confirmed by the FT-IR analysis. The disappearance of C-Br bonds in spectra of FCMP@1-4 compared with monomer 4,4′-dibromooctafluorobiphenyl demonstrated the success of phenyl-acetylene coupling (ESI, Figure S1). The four infrared spectra of the polymers are basically similar and demonstrate two main adsorption regions: a first absorption band in the 650–1250 cm^−1^ region, which is assigned as to benzene ring skeleton vibration; while the second peak close to 2900 cm^−1^, corresponding to –C–H stretching of benzene ring. In addition, a relatively weak peak at approximate 2202 cm^−1^, which referred to –C≡C– stretching of alkynyl moiety of FCMP@1-4, which was further proved that the polymers were synthesized successfully. Elemental analysis indicated that the carbon and hydrogen contents of FCMP@1-4 were close to the theoretical values of an ideal network with a high degree of polycondensation. X-ray diffraction (XRD) showed the amorphous nature of the resulting FCMP@1-4 (ESI, Figure S2a) and FCMP-600@1-4 (ESI, Figure S2b). Transmission electron microscopy (TEM) analyses also showed the amorphous texture of FCMP@1-4 (ESI, Figure S3(a–d)) and FCMP-600@1-4 (ESI, Figure S3(e–h)) materials. Field-emission scanning electron microscopy (FE-SEM; ESI, Figure S4(a–d)) was utilized to investigate the morphology of FCMP@1-4 polymers. The results of FE-SEM show that FCMP@1-4 are irregular sphere shape with particle size 100~300 nm, while FCMP-600@1-4 are irregular lumps with nanometre dimensions (ESI, Figure S4(e–h)). Furthermore, X-ray photoelectron spectroscopy (XPS) results display fluorine elements still exist in FCMP-600@1-4 after pyrolysis (ESI, Figure S5).

**Figure 2:**
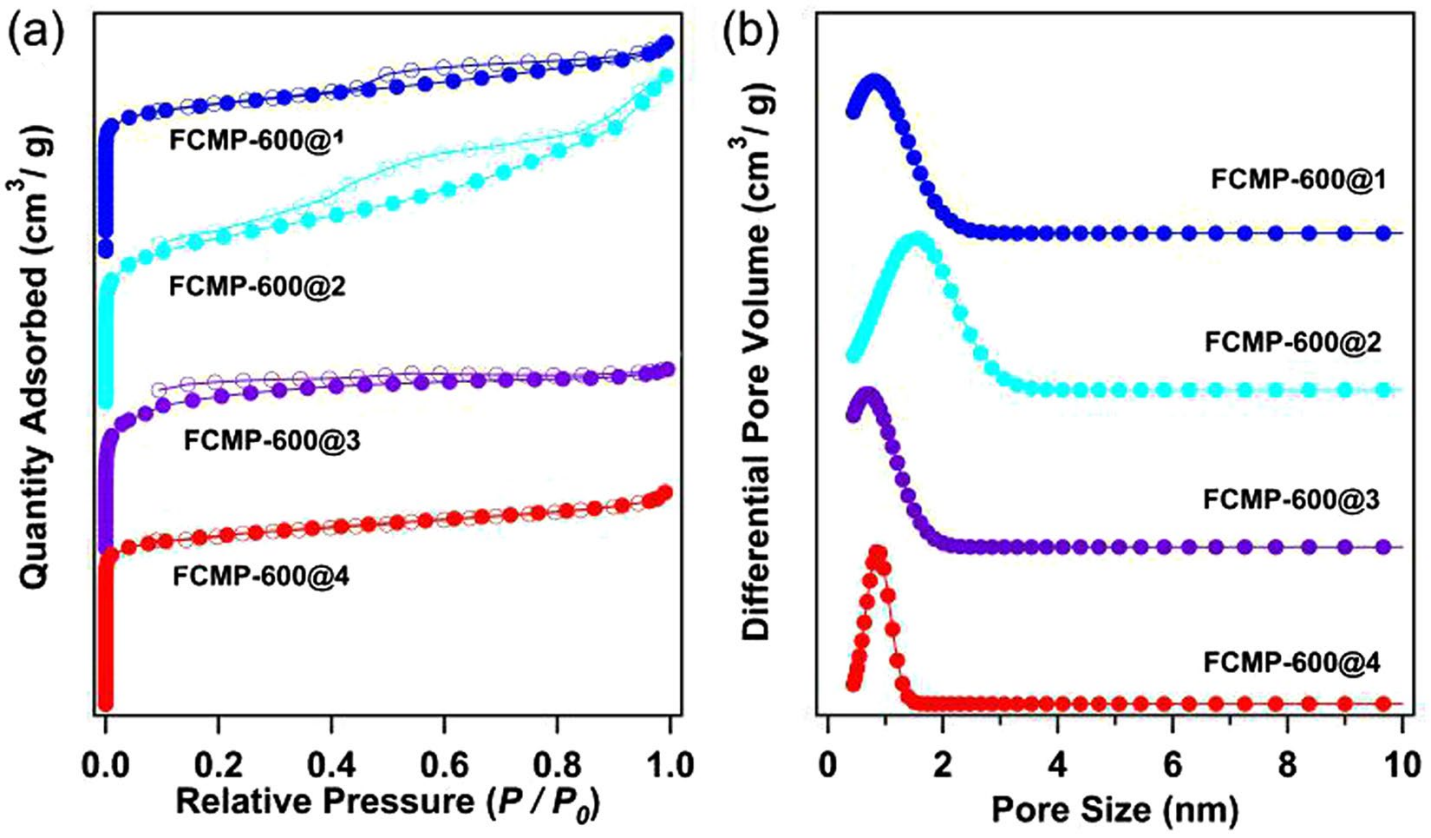
(**a**) Nitrogen adsorption/desorption isotherms measured at 77.3 K for FCMP-600@1–4, the isotherms of FCMP-600@1–3 are shifted vertically by 300, 200 and 100 cm^3^ g^−1^ for better visibility, respectively; (**b**) pore size distributions calculated using density functional theory (DFT) method, for clarity, the curves of FCMP-600@1–3 are shifted vertically by 3, 2 and 1 cm^3^ g^−1^, respectively.

The surface areas and porous properties of FCMP@1-4 and FCMP-600@1-4 were analyzed by nitrogen sorption analysis at 77.3 K. As shown in Figure S6(a–d), except for FCMP@4, the isotherms of FCMP@1, 2 and 3 showed rapid nitrogen adsorption at low pressure. The Brunauer-Emmett-Teller (BET) surface areas of FCMP@1, 2, 3 and 4 were calculated to be 551, 636, 692, and 88 m^2^ g^−1^, respectively. The total pore volumes were 0.3865, 0.6983, 0.4074 and 0.1180 cm^3^ g^−1^, respectively (ESI, Table S1). Compared to FCMP@1-3, FCMP@4 has a significantly low surface area and pore volume. This could be caused by the strong π-π stacking effect between the molecules tetrakis(4-ethynylphenyl)ethene, which lead to formation of planar sheet-like rather than three-dimensional structure^14^. Besides that, the pore size distributions of FCMP@1-4 are very broad (Figure S6(e)). The porosity data of the polymers are summarized in Table S1. In order to overcome this, a successive cross-linking pathway was utilized to improve the BET surface area of the porous polymers. The obtained porous materials FCMP-600@1-4 displayed high surface areas via template-free pyrolysis of FCMPs precursors at 600 °C. The BET surface areas were obtained to be 755, 780, 807 and 901 m^2^ g^−1^ and the total pore volumes were 0.4242, 0.6654, 0.4033 and 0.4331 cm^3^ g^−1^ (micropore volumes calculated from the nitrogen isotherms at *P*/*P*_0_ = 0.0500 are 0.1951, 0.4502, 0.1636 and 0.1998 cm^3^ g^−1^) for FCMP-600@1, FCMP-600@2, FCMP-600@3 and FCMP-600@4, respectively. These results indicated that the surface area and pore volume could be indeed increased by using the pyrolysis of POPs without any templates. As shown in Fig. 2a, FCMP-600@1-4 materials show type I isotherms featured by a sharp uptake at the low-pressure region between *P*/*P*_0_ = 1 × 10^−5^ to 1 × 10^−2^, reflecting the presence of micropores. Distinctly, FCMP-600@1 and 2 possess obvious hysteresis extending to low pressure between the adsorption and desorption isotherms, while FCMP-600@3 displays a relatively tiny hysteresis, which is partly attributed to the swelling in a flexible polymer network, as well as mesopore contribution^11–13^. Compared with FCMP-600@1, 2, and 3, FCMP-600@4 exhibits a negligible hysteresis loop in the whole pressure range, suggesting that this polymer possesses a very rigid molecular structure. The increase in nitrogen sorption at a high relative pressure for FCMP-600@1-4 may arise from the interparticulate porosity associated with the mesopores of the samples. The pore size distributions were calculated from the nonlocal density functional theory (NLDFT) using the model of carbon as an adsorbent, and the main micropore size peaked at 1.05, 1.74, 0.78, and 0.84 nm for FCMP-600@1, 2, 3, and 4, respectively (Fig. 2b).

## Discussion

### Gas uptake capacity and separation

**Figure 3:**
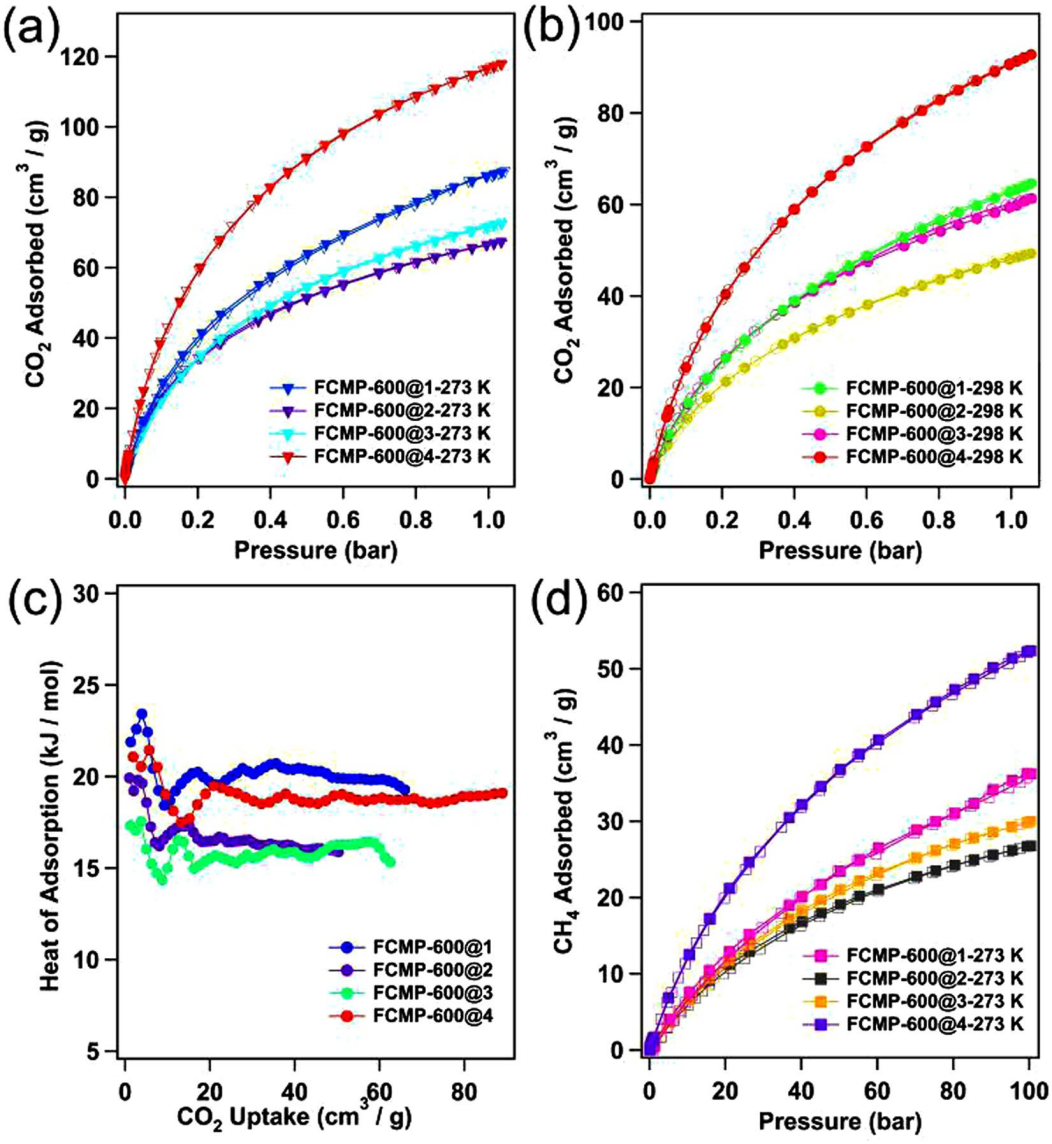
The CO_2_ sorption isotherms of FCMP-600@1-4 collected at (**a**) 273 K and (**b**) 298 K, respectively; (**c**) the isosteric heats of CO_2_ adsorption for FCMP-600@1‒4; (**d**) the CH_4_ sorption isotherms of FCMP-600@1-4 polymers collected at 273 K (solid symbols, adsorption; open symbols, desorption).

The CO_2_ adsorption capacities of FCMP-600@1-4 under 273 K and 298 K were also measured (Fig. 3), which displayed linear trend at both 273 K and 298 K, respectively. At 273 K and 1.05 bar, the CO_2_ capture uptakes of FCMP-600@1, 2, 3, and 4 are 88, 68, 73, and 119 cm^3^ g^−1^ (5.35 mmol g^−1^), respectively (Fig. 3a). The adsorbance also can reach 65, 49, 61 and 93 cm^3^ g^−1^(4.18 mmol g^−1^) for FCMP-600@1, 2, 3, and 4 at 298 K (Fig. 3b). FCMP-600@1-4 can enhance the CO_2_ uptake by 3.3-, 2.3-, 2.7-, and 4.2-fold than those of the corresponding precursor FCMP@1, 2, 3, and 4, respectively (ESI, Figure S7 and Table S1). Among them, FCMP-600@4 dispalys the highest CO_2_ capture capacity than those of other three polymers at both 273 K and 298 K, which could be attributed to the narrower micropore size and higher micropore surface area of FCMP-600@4. This value is a little lower than that of recently reported P-PCz (*S*_BET_ = 1647 m^2^ g^−1^, 5.57 mmol g^−1^)^9^, FCTF^−1^-600 (*S*_BET_ = 1535 m^2^ g^−1^, 5.53 mmol g^−1^)^15^, and PPF^−1^ (*S*_BET_ = 1740 m^2^ g^−1^, 6.12 mmol g^−1^)^16^, but can compete with the best performing POP-based adsorbents like BILP-4 (*S*_BET_ = 1135 m^2^ g^−1^, 5.34 mmol g^−1^)^17^, ALP^−1^ (*S*_BET_ = 1235 m^2^ g^−1^, 5.37 mmol g^−1^)^18^. In particularly, FCMP-600@4 (4.18 mmol g^−1^) also exhibits an excellent CO_2_ capacity at 298 K, which is higher than the reported values for most porous polymers at 273 K, such as CPOP-9 (*S*_BET_ = 2440 m^2^ g^−1^, 4.14 mmol g^−1^)^19^, CPOP-8 (*S*_BET_ = 1610 m^2^ g^−1^, 3.75 mmol g^−1^)^18^, and *fl*-CTF400 (*S*_BET_ = 2862 m^2^ g^−1^, 4.13 mmol g^−1^)^20^. Compared to FCMP-600@1 and 3, FCMP-600@2 has a broader micropore size distribution. Therefore, FCMP-600@2 exhibits lower CO_2_ capture capacity than those of FCMP-600@1 and 3 at the same conditions, although FCMP-600@1, 2 and 3 show the similar BET surface areas.

The isosteric heat (*Q*_st_) of adsorption CO_2_ was estimated from adsorption data collected under 273 K and 298 K through the Clausius-Clapeyron equation. At zero coverage, the *Q*_st_ of FCMP-600@1, 2, 3, and 4 are 23.4, 19.9, 17.3, and 21.4 kJ mol^−1^, respectively (Fig. 3c). The *Q*_st_ are lower than the values reported for imine-linked organic polymers, CTFs, diimide polymers, and so on^9,15–20^. The relatively high CO_2_ uptake and binding by FCMP-600@1-4 are most likely due to favorable interactions of the polarizable CO_2_ molecules through hydrogen bonding and/or dipole quadrupole interactions that utilize the proton-free fluorine sites of phenyl rings^21–25^.

In light of high CO_2_ capture capacities, high surface areas, fluorine-enriched skeletons, and small pore sizes for FCMP-600@1-4, it is reasonable to study the selective uptake of FCMP-600@1-4 for small gases (CO_2_, CH_4_, and N_2_) to evaluate their potential use in gas separation. The methane isotherms depicted in Fig. 3d are fully reversible and the uptakes of FCMP-600@1, 2, 3, and 4 reach 36, 27, 30, and 53 cm^3^ g^−1^ at 273 K and 1.0 bar, respectively (ESI, Figure S8 and Table S1). The CH_4_ uptakes of FCMP@1-4 were 4.8-6.6 cm^3^ g^−1^ at the same conditions (ESI, Figure S9 and Table S1). Apparently, FCMP-600@1-4 are higher 4.5^−1^1 times than those of the FCMP@1-4 precursors. This result implyed that the FCMP-600@1-4 can efficiently capture CH_4_ due to high microsurface area and micropore volume^26,27^. The selectivities of FCMP-600@1-4 toward CO_2_ over CH_4_ and N_2_ were investigated by collecting pure component physisorption isotherms at 273 K (ESI, Figure S8), and then which were predicted from the experimental pure component isotherms using the ideal adsorbed solution theory (IAST). At zero coverage, the high CO_2_/N_2_ selectivity was recorded for FCMP-600@1-4 (109-77 at 273 K) (ESI, Figure S10a). Moreover, FCMP-600@1-4 show a moderate level CH_4_/N_2_ selectivities: 8–11 (273 K) (ESI, Figure S10b).

### Iodine capture

**Figure 4:**
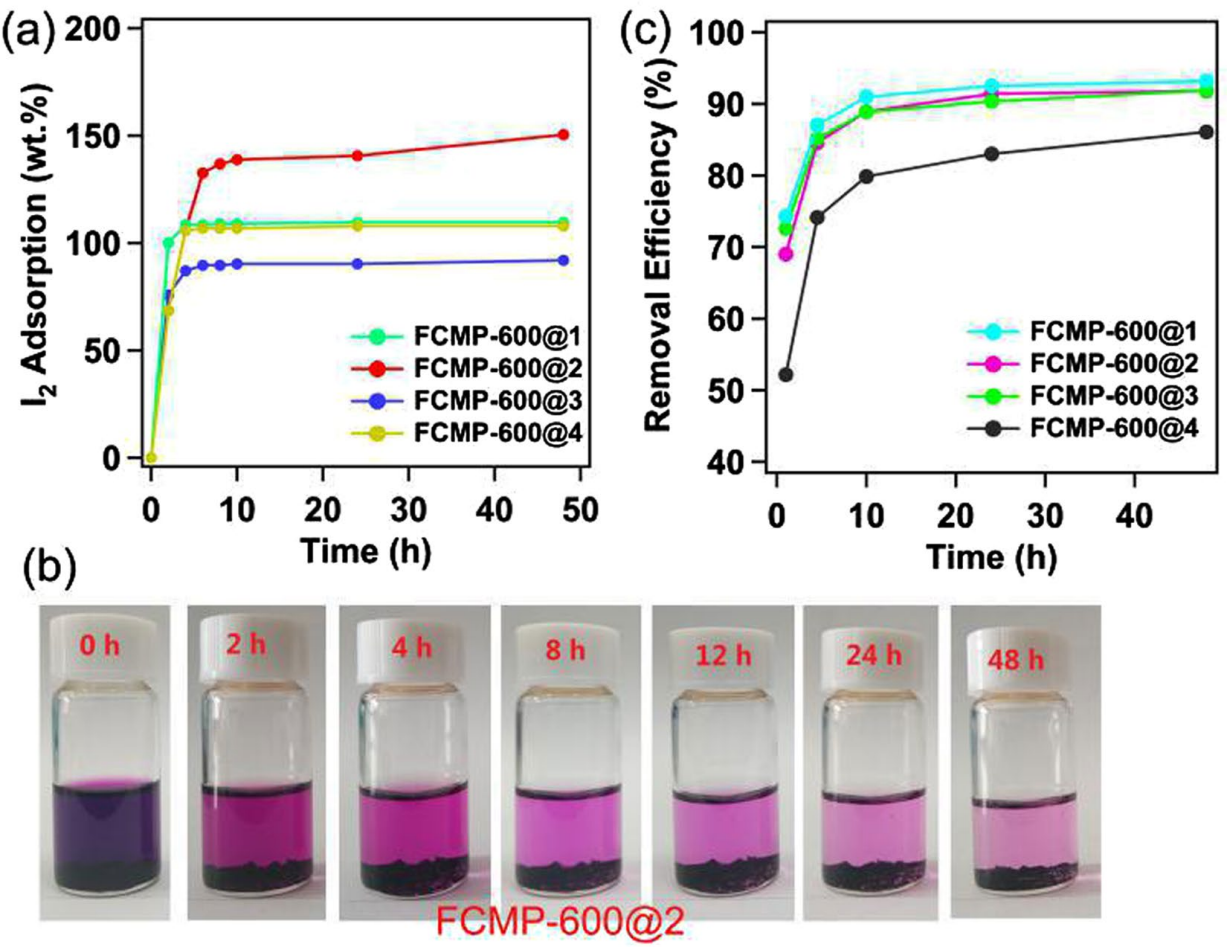
(**a**) Gravimatric uptake of iodine as a function of time at 350 K; (**b**) the photograph of FCMP-600@2 shows the different iodine adsorption rates; (**c**) the kinetic studies of iodine absorbance by FCMP-600@1-4 in hexane solution at room temperature (4 mg mL^−1^).

In the recent years, the capture of iodine using porous materials has attracted considerable interest. Most interestingly, we found the fluorine-enriched polymers were highly efficient for the iodine adsorption. The absorption of solid iodine was conducted by exposing the samples to nonradioactive iodine vapor in a sealed vessel at 350 K and ambient pressure, which was the typical fuel reprocessing condition. Gravimetric measurement was performed at different time intervals during the iodine loading (Fig. 4a). Except for FCMP-600@2, the maximum iodine uptakes of other three porous materials were reached quickly saturated in the first 4 h. As the synthesized polymers, FCMP-600@2 has the maximum value for iodine uptake reached up to 141 wt.%, followed by FCMP-600@4 (111 wt.%), FCMP-600@1 (108 wt.%), and FCMP-600@3 (90 wt.%). The thermogravimetric analysis (TGA) of the I_2_-loaded FCMP-600@2 and 4 polymers reveal a significant weight loss from 90 to 300 °C (ESI, Figure S11), the calculated iodine mass loss were 152 and 105 wt.% for FCMP-600@2 and FCMP-600@4, respectively, which was close to the saturated adsorption value.

Additionally, the FCMP-600@1-4 are capable of capture iodine in solution. When the FCMP-600@2 (30 mg) in iodine/hexane solution (4 mg mL^−1^, 3 mL), the dark purple solution gradually faded to light purple (Fig. 4b and Figure S12). The UV/Vis absorption intensity of the samples was decreased with the prolonged action time (ESI, Figure S13). It can be observed from the adsorption kinetics of iodine at room temperature that the adsorption process was affected by the contact time (Fig. 4c). In the initial stage, the adsorption capacity increased quickly with the prolonged contact time, and then slow down to equilibrium after about 10 h. The removal efficiencies of polymers achieved for the solution are 81.2–92.4%. The adsorption kinetics of iodine for FCMP-600@1-4 were analyzed through the frequently used pseudo-first-order and pseudo-second-order models were adopted^22^. Results show that the adsorption data fits well in pseudo-second-order kinetic model with good linear correlation coefficient (*R* ^2^) values of 0.9961, 0.9962, 0.9962 and 0.9962 for iodine solution of FCMP-600@1, 2, 3 and 4, respectively (ESI, Table S2, Figures S14 and S15). This confirmed that the iodine adsorption process in this work was governed by the pseudo-second-order kinetics. The XPS spectrum of fluorine-enriched polymers indicated that the coexistence of elemental iodine and triiodide ion, which suggested a hybrid of physisorption and chemisorption (ESI, Figure S16). Furthermore, it is very easy to remove or release the trapped iodine molecules of the samples via immersion of the iodine-loaded sample in ethanol. When the I_2_-FCMP-600@1-4 were immersed in ethanol, the colour of the solvent were changed from colourless to dark brown (ESI, Figure S17), indicating that the iodine guests were released from the solid. The four samples were recycled easily for at least five times without significant loss of iodine uptake (ESI, Figure S18).

The saturated iodine adsorption capacities of FCMP-600@1-4 can be determined from the adsorption isotherms (ESI, Table S3, Figures S19 and S20). Two different adsorption stages were observed from the plot of the equilibrium concentration versus the quantities of the adsorbed iodine at equilibrium. At first, the equilibrium uptake increases linearly with the increase of iodine solution concentration at low concentration. Then, the adsorption reached its maximum value and the adsorption process turned to be independent on the concentration. The simulation results revealed that the iodine adsorption of samples could be well described using Langmuir adsorption isotherm (ESI, Figures S19 and S20), suggesting a monolayer adsorption behavior for iodine molecule on the surface of polymers. From the sorption kinetics, the maximum capacities for iodine uptake reached up to around 550, 729, 520, and 539 mg g^−1^ for FCMP-600@1, 2, 3, and 4, respectively.

## Conclusion

In summary, four the novel fluorine-enriched porous materials were successfully designed and synthesized. The properties of FCMP-600@1-4 were well investigated and discussed. The BET surface areas of FCMP-600@1-4 can be tuned by changing the geometry and size of comonomer. FCMP-600@1-4 have the BET specific surface areas of 755-901 m^2^ g^−1^ as well as permanent microporosity, and the abundant fluorine atoms in the skeleton endow the materials with high CO_2_/N_2_ (109–77) and CH_4_/N_2_ (8^−1^1) selectivities. At 273 K and 1.05 bar, FCMP-600@4 exhibits the highest CO_2_ uptake of 119 cm^3^ g^−1^, and CH_4_ uptake of 53 cm^3^ g^−1^, and the rest materials are in the range of 68-88 cm^3^ g^−1^ for CO_2_. Meanwhile, FCMP-600@1-4 show good adsorption capacities of 90–141 wt.% toward iodine vapor. We hope this type of fluorine-doped absorbent can be effective for gas storage and will bring new application possibilities.

## Methods

### Synthesis of FCMP@1

4,4′-Dibromooctafluorobiphenyl (114 mg, 0.25 mmol) and 1,3-diethynylbenzene (47 mg, 0.375 mmol) were put into a 50 mL two-necked round-bottom flask, then the flask exchanged 3 cycles under vacuum/N_2_. Then added to 2 mL DMF and 2 mL triethylamine (Et_3_N), the flask was further degassed by the freeze-pump-thaw for 3 times. When the solution had reached reaction temperature, a slurry of tetrakis(triphenylphosphine)palladium(0) (19.9 mg, 0.017 mmol) in the 1 mL DMF and copper(I) iodine (3.1 mg, 0.017 mmol) in the 1 mL Et_3_N was added, and the reaction was stirred at 120 °C for 48 h under nitrogen atmosphere. The solid product was collected by filtration and washed well with THF, methanol, acetone, and water for 4 times, respectively. Further purification of the polymer was carried out by Soxhlet extraction with methanol, and THF for 24 h, respectively, to give **FCMP@1** as yellow powder (88.7% yield). Elemental Analysis (%) C 69.24, H 1.55. Found: C 66.88, H 1.16.

### Synthesis of FCMP@2

4,4′-Dibromooctafluorobiphenyl (114 mg, 0.25 mmol) and 1,3,5-triethynylbenzene (45 mg, 0.25 mmol) were put into a 50 mL two-necked round-bottom flask, then the flask exchanged 3 cycles under vacuum/N_2_. Then added to 2 mL DMF and 2 mL triethylamine (Et_3_N), the flask was further degassed by the freeze-pump-thaw for 3 times, purged with N_2_. When the solution had reached reaction temperature, a slurry of tetrakis(triphenylphosphine)palladium(0) (27.7 mg, 0.024 mmol) in the 1 mL DMF and copper(I) iodine (5.7 mg, 0.032 mmol) in the 1 mL Et_3_N was added, and the reaction was stirred at 120 °C for 48 h under nitrogen atmosphere. The solid product was collected by filtration and washed well with THF, methanol, acetone, and water for 4 times, respectively. Further purification of the polymer was carried out by Soxhlet extraction with methanol, and THF for 24 h, respectively, to give **FCMP@2** as brown solid (93.6% yield). Elemental Analysis (%) Calcd. (Actual value for an infinite 2D polymer) C 70.86, H 1.11. Found: C 68.17, H 0.95.

### Synthesis of FCMP@3

4,4′-Dibromooctafluorobiphenyl (114 mg, 0.25 mmol) and tetrakis(4-ethynylphenyl)methane (78 mg, 0.188 mmol) were put into a 50 mL two-necked round-bottom flask, then the flask exchanged 3 cycles under vacuum/N_2_. Then added to 2 mL DMF and 2 mL triethylamine (Et_3_N), the flask was further degassed by the freeze-pump-thaw for 3 times. When the solution had reached reaction temperature, a slurry of tetrakis(triphenylphosphine)palladium(0) (17.9 mg, 0.015 mmol) in the 1 mL DMF and copper(I) iodine (3.7 mg, 0.02 mmol) in the 1 mL Et_3_N was added, and the reaction was stirred at 120 °C for 48 h under nitrogen atmosphere. The solid product was collected by filtration and washed well with THF, methanol, acetone, and water for 4 times, respectively. Further purification of the polymer was carried out by Soxhlet extraction with methanol, and THF for 24 h, respectively, to give **FCMP@3** as yellowish-brown powder (94.3% yield). Elemental Analysis (%) C 82.44, H 3.07. Found: C 78.28, H 3.43.

### Synthesis of FCMP@4

4,4′-Dibromooctafluorobiphenyl (114 mg, 0.25 mmol) and 1,1,2,2-tetrakis(4-ethynylphenyl)ethene (71.5 mg, 0.188 mmol) were put into a 50 mL two-necked round-bottom flask, then the flask exchanged 3 cycles under vacuum/N_2_. Then added to 2 mL DMF and 2 mL triethylamine (Et_3_N), the flask was further degassed by the freeze-pump-thaw for 3 times. When the solution had reached reaction temperature, a slurry of tetrakis(triphenylphosphine)palladium(0) (17 mg, 0.015 mmol) in the 1 mL DMF and copper(I) iodine (2.7 mg, 0.015 mmol) in the 1 mL Et_3_N was added, and the reaction was stirred at 120 °C for 48 h under nitrogen atmosphere. The solid product was collected by filtration and washed well with THF, methanol, acetone, and water for 4 times, respectively. Further purification of the polymer was carried out by Soxhlet extraction with methanol, and THF for 24 h, respectively, to give **FCMP@4** as pale yellow powder (88.7% yield). Elemental Analysis (%) C 82.83, H 3.01. Found: C 75.79, H 2.12.

### Synthesis of FCMP-600@1-4

The pyrolysis reactions of the **FCMP@1-4** were carried out on quartz tubes in an electric furnace under argon atmosphere. The FCMP@1, 2, 3, and 4 samples were heated from the room temperature to 600 °C with a heating rate of 3 °C/min, then pyrolyzed at 600 °C for 2 h in argon gas (400 sccm), respectively. The pyrolysis reactions at 600 °C in argon gas were denoted to **FCMP-600@1**, **FCMP-600@-2**, **FCMP-600@-3**, and **FCMP-600@4**, respectively.

## Electronic supplementary material

Supplementary Information
