# Supplementary material for: Synthesis of tunable porosity of fluorine-enriched porous organic polymer materials with excellent CO2, CH4 and iodine adsorption
Source: Sci Rep. 2017 Oct 25;7:13972. doi: 10.1038/s41598-017-14598-0 (PMC5656616; doi:10.1038/s41598-017-14598-0)
Supplement: Supplementary file 1 — Supplementary Information [file 41598_2017_14598_MOESM1_ESM.pdf]

Supporting Information

Synthesis of tunable porosity of fluorine-enriched porous organic polymer materials with excellent CO_2, CH_4 and iodine adsorption

Guoyan Li,[a] Chan Yao,[a] Jiku Wang[a] and Yanhong Xu[a,b]*

Corresponding Author:
Professor Yan-Hong Xu
[a]*Key Laboratory of Preparation and Applications of Environmental Friendly Materials (Jilin Normal University), Ministry of Education, Changchun, 130103, China*
[b]*Key Laboratory of Functional Materials Physics and Chemistry of the Ministry of Education, Jilin Normal University, Siping 136000, China*
Email: xuyh198@163.com

Contents

Section A. Materials and methods

Section B. Synthetic procedures

Section C. FT-IR spectral profiles

Section D.PXRD curves

Section E. HR-TEM images

Section F. SEM images

Section G. F1s XPS spectra

Section H. Nitrogen sorption isotherm of FCMP@1-4

Section I. Gas adsorption isotherms

Section J. Gas selectivity analyses

Section K. Iodine capture analyses

Section L. Supporting references

Section A. Materials and methods

4,4'-Dibromooctafluorobiphenyl was purchased from Alfa. 1,3,5-Triethynylbenzene and 1,3-diethynylbenzene were purchased from TCI. Tetrakis(triphenylphosphine)palladium(0), copper(I) iodide. All the solvents used were purchased from Aladdin.

^{1}H NMR spectra were recorded on Bruker Avance III models HD 400NMR spectrometers, where chemical shifts (δ in ppm) were determined with a residual proton of the solvent as standard. Fourier transform Infrared (FT-IR) spectra were recorded on a Perkin-elmer model FT-IR-frontier infrared spectrometer. The solution UV-visible analyzer was used for shimadzu UV-3600. X-ray photoelectron spectra (XPS) were recorded on an ESCALAB250Xi electron spectrometer (Thermo Fisher Scientific Inc., Waltham, MA, USA). Field-emission scanning electron microscopy (FE-SEM) images were performed on a JEOL model JSM-6700operating at an accelerating voltage of 5.0 kV. The samples were prepared by drop-casting a THF suspension onto mica substrate and then coated with gold. High-resolution transmission electron microscopy (HR-TEM) images were obtained on a JEOL model JEM-3200 microscopy. Powder X-ray diffraction (PXRD) data were recorded on a Rigaku model RINT Ultima III diffractometer by depositing powder on glass substrate, from $2\theta = 1.5°$ up to 60° with 0.02° increment. The elemental analysis was carried out on a EuroEA-3000.TGA analysis was carried out using a Q5000IR analyser (TA Instruments) with an automated vertical overhead thermobalance. Before measurement, the samples were heated at a rate of 5 °C min^{-1} under a nitrogen atmosphere.

Nitrogen sorption isotherms were measured at 77 K with ASIQ (iQ-2) volumetric adsorption analyzer. Before measurement, the samples were degassed in vacuum at 150 °C for more than 10 h. The Brunauer-Emmett-Teller (BET) method was utilized to calculate the specific surface areas and pore volume. The nonlocal density functional theory (NLDFT) was applied for the estimation of pore size and pore size distribution.

Carbon dioxide sorption isotherms were measured at 298 K and 273 K with a Bel Japan Inc. model BELSORP-max analyzer, respectively. Before measurement, the samples were also degassed in vacuum at 120 °C for more than 10 h.

Methane and nitrogen sorption isotherms were measured at 273 K with a Bel Japan Inc. model BELSORP-max analyzer, respectively. Before measurement, the samples were also degassed in vacuum at 120 °C for more than 10 h.

Section B. Synthetic procedures

Synthesis of tetrakis(4-((trimethylsilyl)ethynyl)phenyl)methane[S1]

Tetra(4-bromophenyl)methane (2.04 g, 3.16 mmol), $PdCl_2(PPh_3)_2$ (0.135 g, 0.18 mmol), CuI (0.024 g, 0.125 mmol), and PPh_3 (0.1 g, 0.38 mmol) were put into a 250 mL round-bottom flask; then the flask exchanged 3 cycles under vacuum/N_2, anhydrous iPr_2NH (30 mL) and trimethylsilylacetylene (2.2 mL, 30.37 mmol) was added via a syringe under the N2. The reaction mixture was brought to reflux at 90°C for 24 h, and then cooled down to room temperature. Solvent was removed in vacuum, and $CHCl_3$ was added to dissolve the residue and filtered through a pad of Celite. The filtrate was washed with dilute Na_2EDTA solution, and then dried over anhydrous Na_2SO_4; the solution was concentrated, and ethanol was added to obtain tetra(4-trimethylsilyacetylenephenyl)methane as a white solid product (80% yield). ^1H NMR ($CDCl_3$, 400 MHz): δ (ppm) 7.26 (d, 8H), 6.85 (d, 8H), 0.26 (s, 36H).

Synthesis of tetrakis(4-ethynylphenyl)methane[S1]

NaOH (0.98 g, 24.6 mmol) was dissolved in 10 mL of CH_3OH, then added to a solution of tetrakis(4-((trimethylsilyl)ethynyl)phenyl)methane (1.8 g, 1.2 mmol) in 20 mL of CH_2Cl_2, and then stirred for 6 h at room temperature. The reaction mixture was washed with water, and the aqueous phase was extracted with CH_2Cl_2, and the combined organic phases were washed with brine, and then dried over anhydrous Na_2SO_4. The solution was concentrated, and

ethanol was added to the solution. Tetrakis(4-ethynylphenyl)methane was obtained as light yellow solid (83% yield). ^{1}H NMR ($CDCl_3$, 400 MHz): δ (ppm) 7.39 (d, 8H),7.12(d, 8H), δ3.06 (s, 4H)

Synthesis of *p*-tetrabromotetraphenylethene (TBTPE) [S2]

Powdered 1,1,2,2-tetraphenylethene (5.00 g, 15.0 mmol) was treated with bromine (7.50 mL, 0.15 mol) and the mixture was kept for 16 h at room temperature. The resulting solid was dissolved in hot toluene (120 mL), concentrated to about 20 mL, and the precipitate was isolated. Purification using flash chromatography on SiO_2 (hexanes/CH_2Cl_2, 20:1 in vol.) gives TBTPE as a colorless solid (5.94 g) in 61% yield. ^{1}H NMR ($CDCl_3$, 400 MHz): *δ* (ppm) 7.26 (d, 8H), 6.85 (d, 8H).

Synthesis of 1,1,2,2-tetrakis[4-(trimethylsilylethynyl)phenyl]ethene[S3]

TBTPE (1g, 1.54 mmol) and $PdCl_2(PPh_3)_2$ (25.2 mg, 0.036 mmol) and CuI (3.6 mg, 0.0185 mmol) were put in to a 100 mL round-bottom flask, then the flask exchanged 3 cycles under vacuum/N_2, then added to 50 mL diethylamine. The flask was degassed by freeze-pump-thaw for 3 times, then warmed to room temperature, refilled with N_2, trimethylsilylacetylene (1 mL, 7.392 mmol) was slowly added via a syringe. The mixture was heated at 50 °C for 15 h. After this the reaction mixture was cooled to room temperature, concentrated to about 10 mL. The crude product was purified by silica gel column chromatography using hexane as eluent. A white solid was obtained (72% yield). ^{1}H NMR ($CDCl_3$, 400 MHz): *δ* (ppm): 7.23 (d, 8H), 6.92 (d, 8H), 0.26 (s, 36H).

Synthesis of 1,1,2,2-tetrakis(4-ethynylphenyl)ethene[S3]

1,1,2,2-Tetrakis(4-(trimethylsilylethynyl)phenyl)ethene (1.5 g, 2.1 mmol) and THF (40 mL) were placed were put into a 250 mL round-bottom flask. Then, KOH (1.8 g, 32 mmol) dissolved in 40 mL of methanol was added. The mixture was stirred at room temperature

overnight. After most of the solvent was evaporated, 100 mL of 1 M aqueous HCl solution was added and the mixture extracted with dichloromethane three times. The organic phases were combined and washed with water and brine and then dried over $MgSO_4$. After filtration and solvent evaporation, the crude product was purified by a silica gel column chromatography using hexane/dichloromethane (100:1 by volume) mixture as eluent. A yellow solid was obtained (81% yield). ^1H NMR (400 MHz, $CDCl_3$): δ (ppm) 7.28 (d, 8H), 6.93 (d, 8H), 3.06 (s, 4H).

Table S1 | Porosity properties and gas uptake for the polymers.

**Polymers**	$S_{BET}{}^{a}$ /m^2 g^{-1}	$S_{micro}{}^{b}$ /m^2 g^{-1}	$V_{total}{}^{c}$ /cm^3 g^{-1}	V_{micro} /cm^3 g^{-1}	CO_2 at 273 K/cm^3 g^{-1}	CO_2 at 298 K/cm^3 g^{-1}	CH_4 at 273 K/cm^3 g^{-1}
**FCMP@1**	551	356	0.3865	0.2277	26	-	5.5
**FCMP@2**	636	241	0.6983	0.5877	29	-	6.0
**FCMP@3**	692	490	0.4074	0.1940	27	-	6.6
**FCMP@4**	88	31	0.1180	0.0918	28	-	4.8
**FCMP-600@1**	755	596	0.4242	0.1951	88	65	36
**FCMP-600@2**	780	508	0.6654	0.4502	68	49	27
**FCMP-600@3**	807	642	0.4033	0.1636	73	61	30
**FCMP-600@4**	975	697	0.4347	0.1851	119	93	53

[a]Brunauer-Emmett-Teller surface area. [b]Total pore volume determined from the N_2 isotherm at P/P_0 =0.995. [c]Micro-pore volume determined from the N_2 isotherm at P/P_0 = 0.050.

Section C. FT-IR Spectra

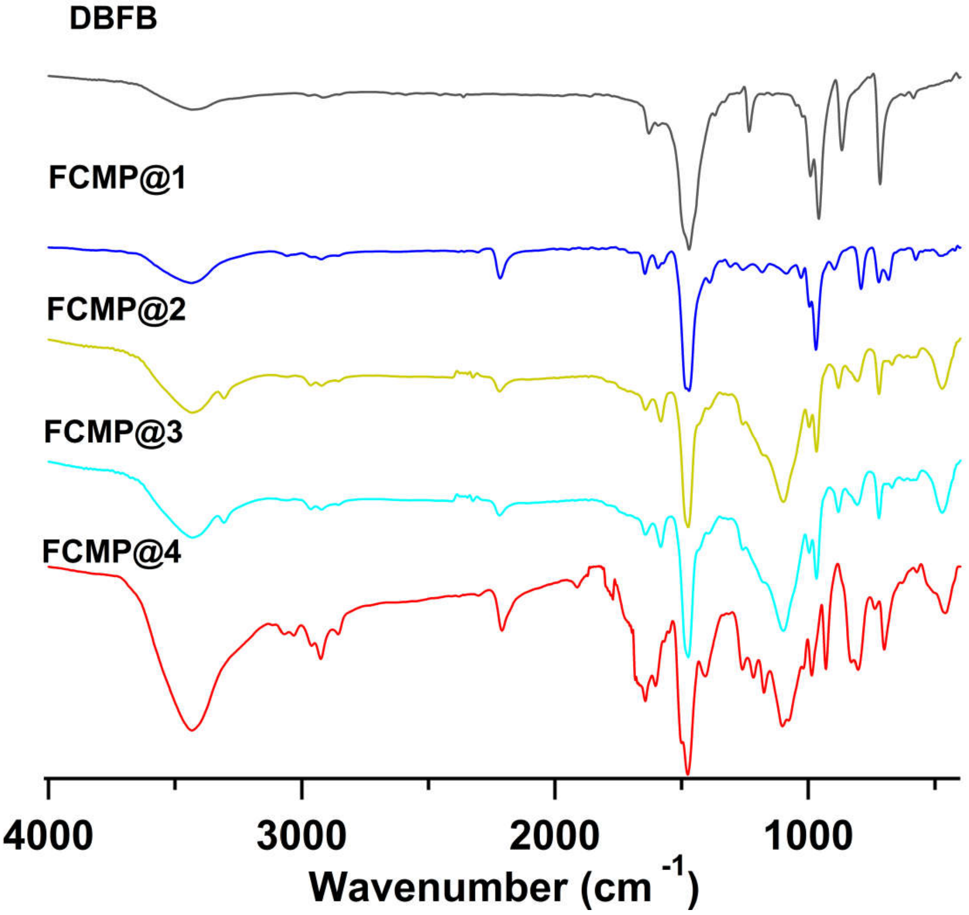

Figure S1 | FT-IR spectra of the FCMP@1-4 porous polymers.

Section D. PXRD curves

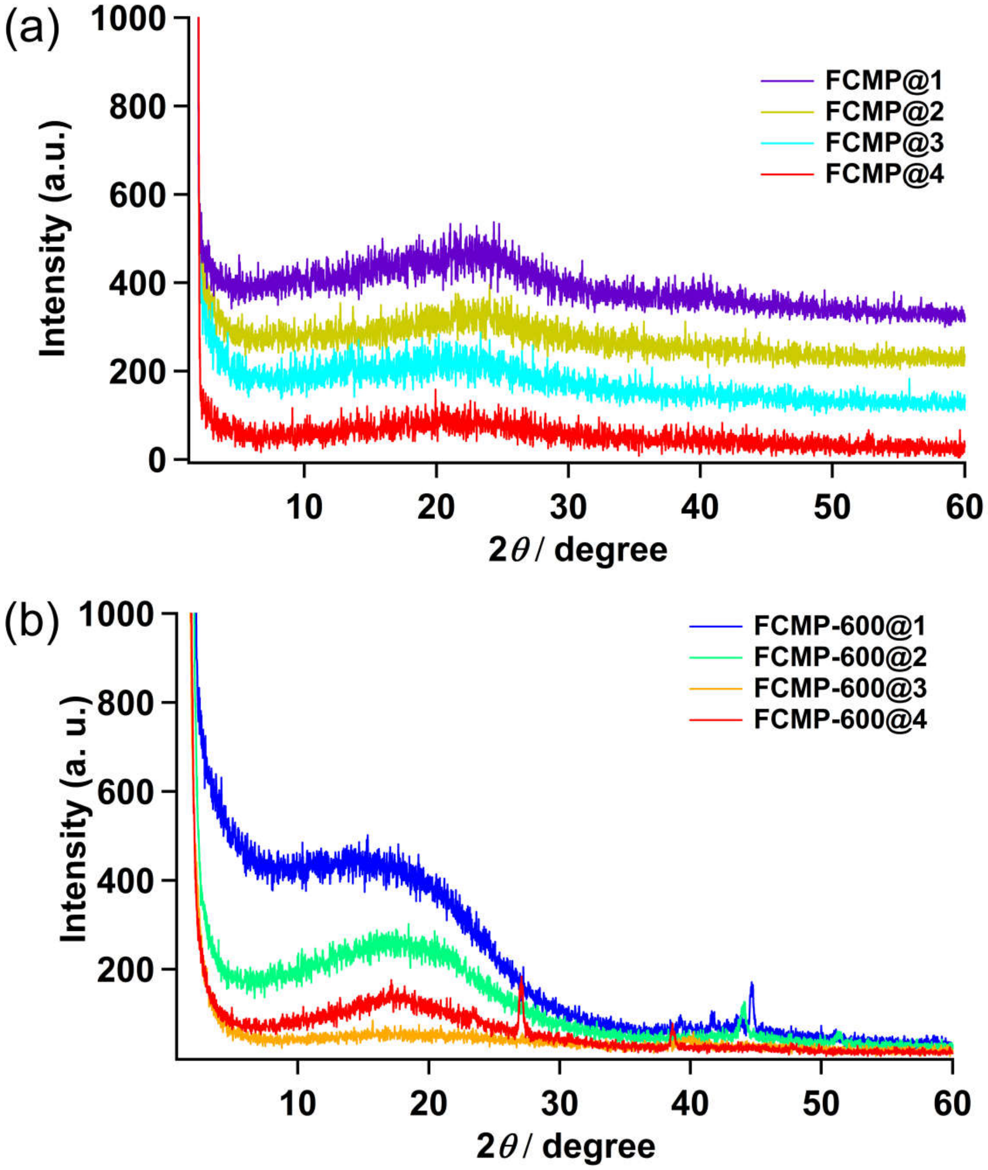

Figure S2 | Powder X-ray diffraction profiles of (a)FCMP@1-4, (b) FCMP-600@1-4.

Section E. HR-TEM images

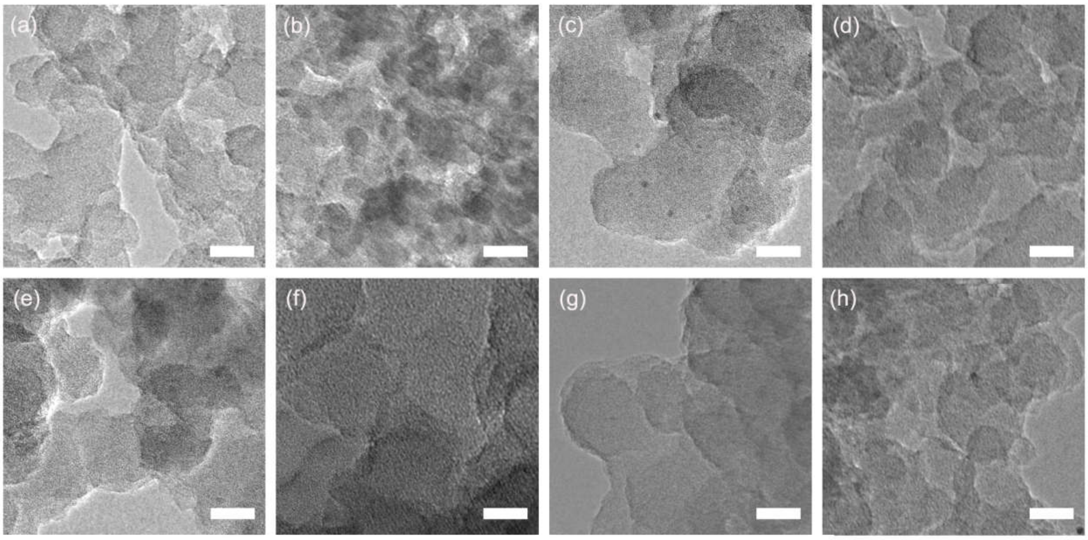

Figure S3 | HR-TEM images of (a) FCMP@1, (b) FCMP@2, (c) FCMP@3, (d) FCMP@4, (e) FCMP-600@1, (f) FCMP-600@2, (g) FCMP-600@3, and (h) FCMP-600@4 (scale bar 20 nm), respectively.

Section F. SEM images

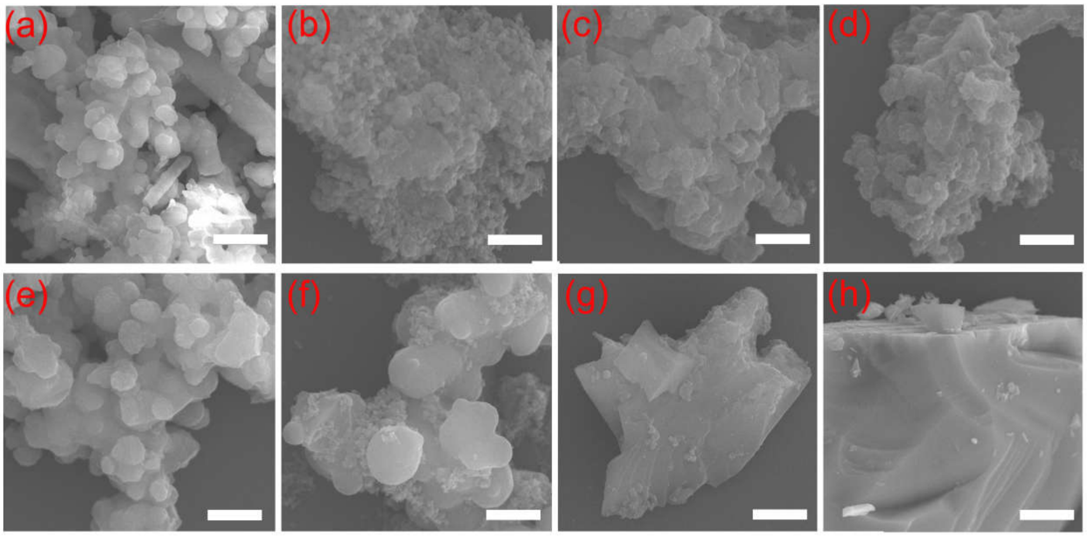

Figure S4 | FE-SEM images of (a) FCMP@1, (b) FCMP@2, (c) FCMP@3, (d) FCMP@4, (e) FCMP-600@1, (f) FCMP-600@2, (g) FCMP-600@3, and (h) FCMP-600@4 (scale bar 1μm), respectively.

Section G. F1s XPS spectra

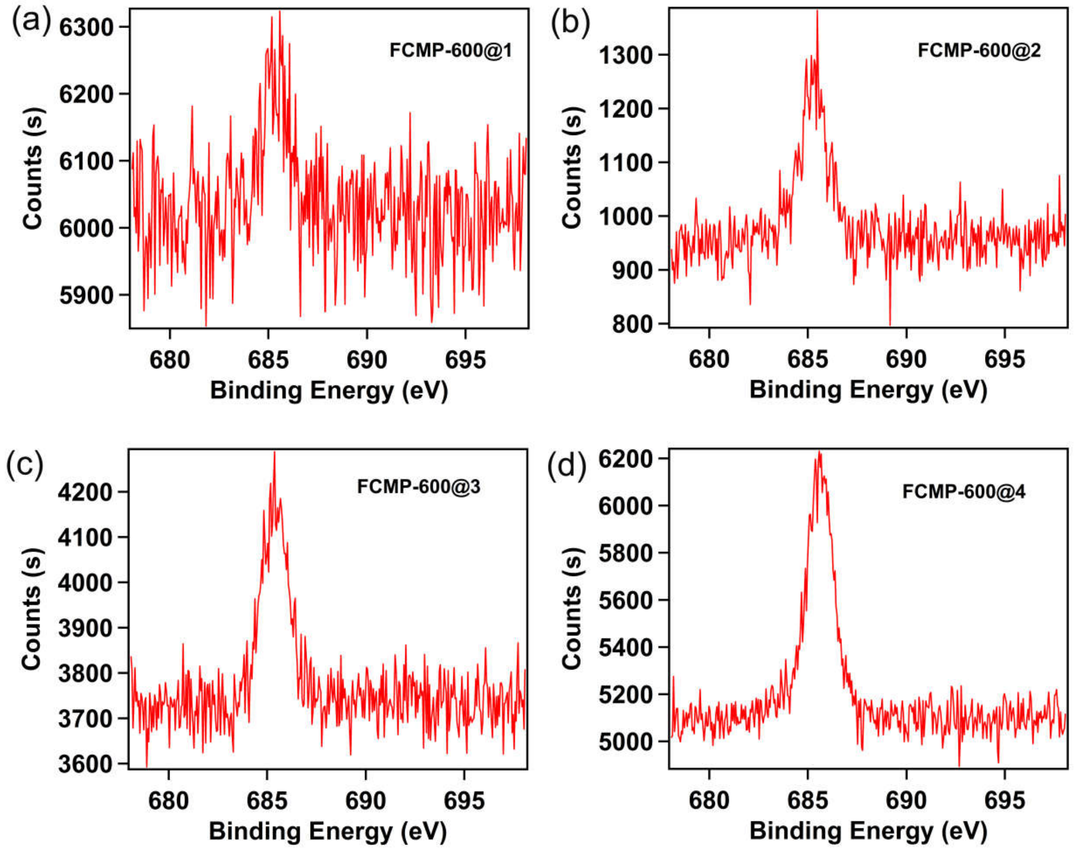

Figure S5 | F1s XPS spectra of (a) FCMP-600@1, (b) FCMP-600@2, (c) FCMP-600@3, and (d) FCMP-600@4, respectively.

Section H. Nitrogen sorption isotherm of FCMP@1-4

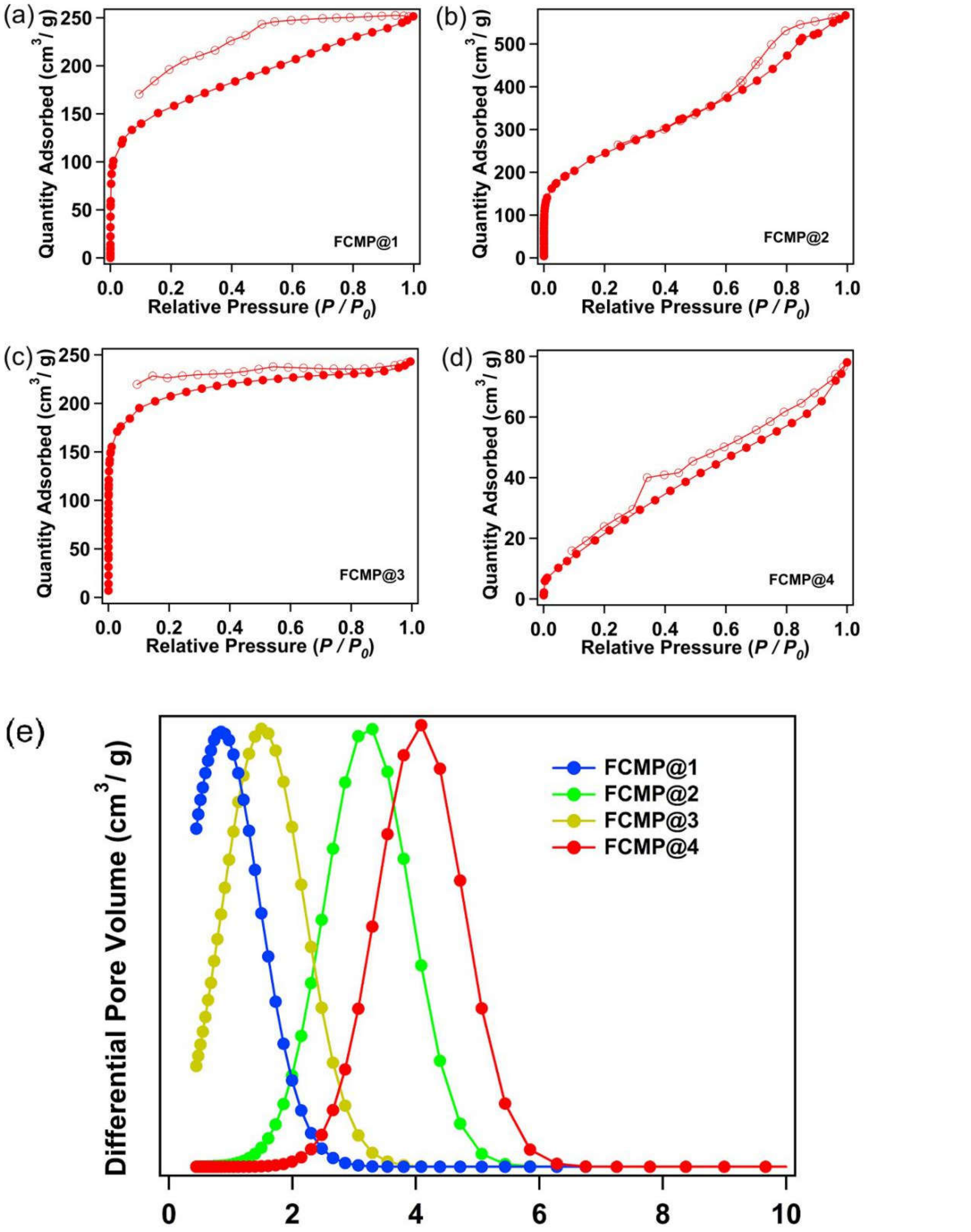

Figure S6 | (a-d) Nitrogen sorption isotherms measured at 77.3 K for FCMP@1–4; (e) pore size distributions calculated using density functional theory (DFT) method.

SectionI. Gas adsorption isotherms

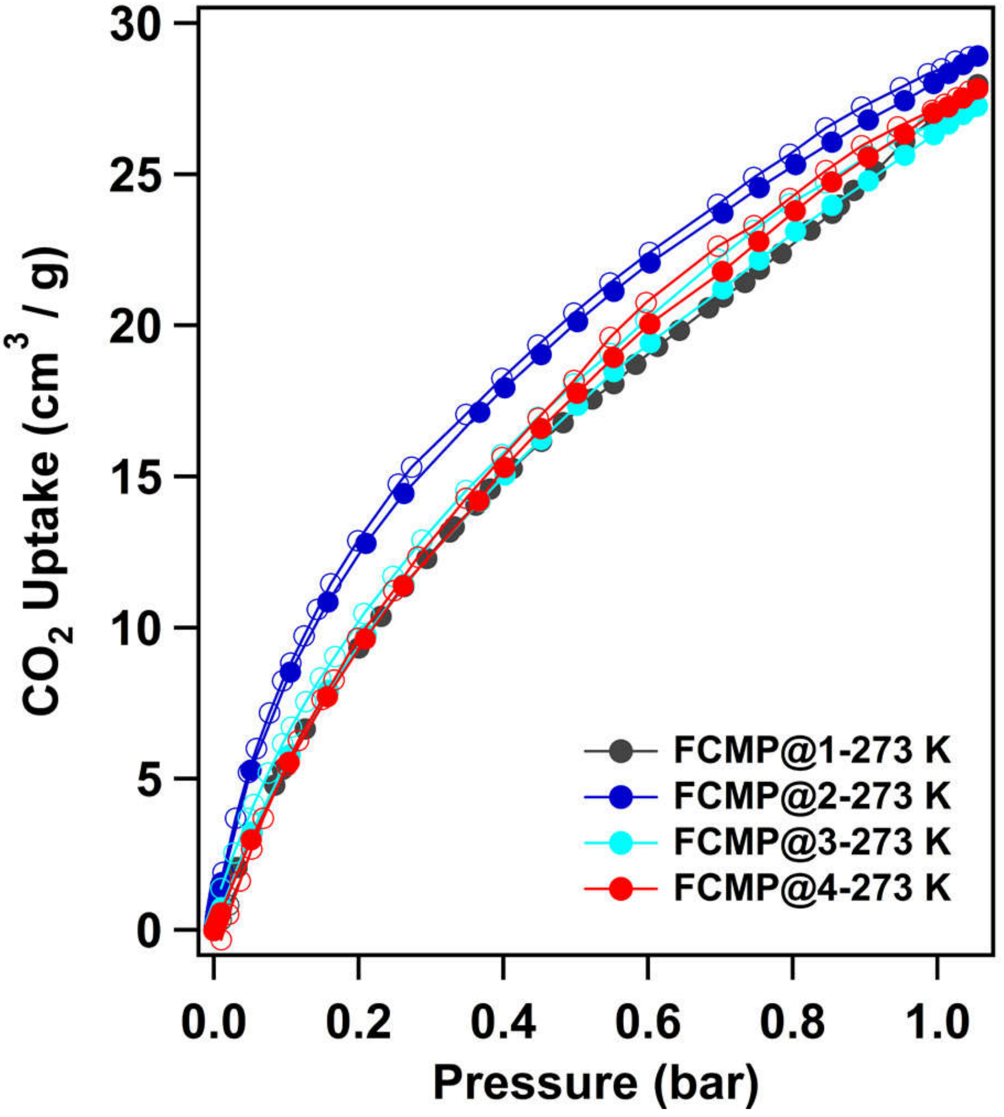

Figure S7 | CO_2 uptake of polymers FCMP@1-4 at 273 K and 1.05 bar.

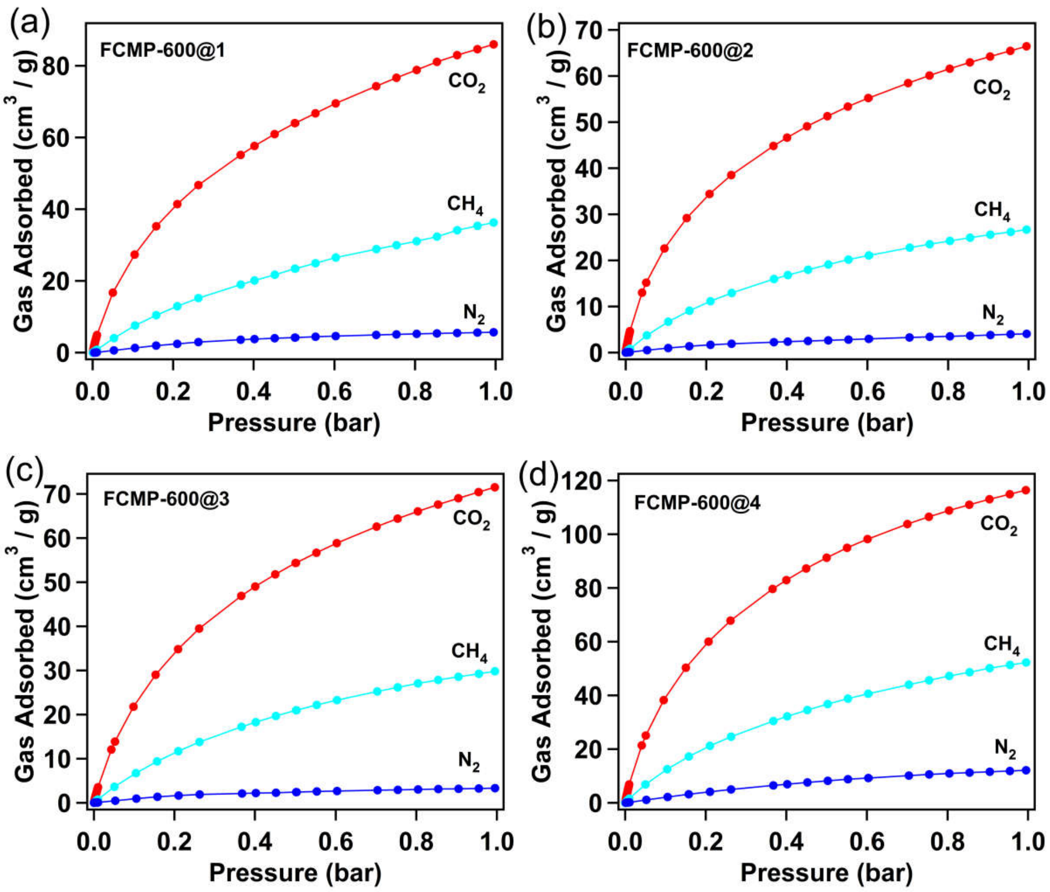

Figure S8 | Gas adsorption isotherms of polymers FCMP-600@1-4 at 273 K and 1.05 bar.

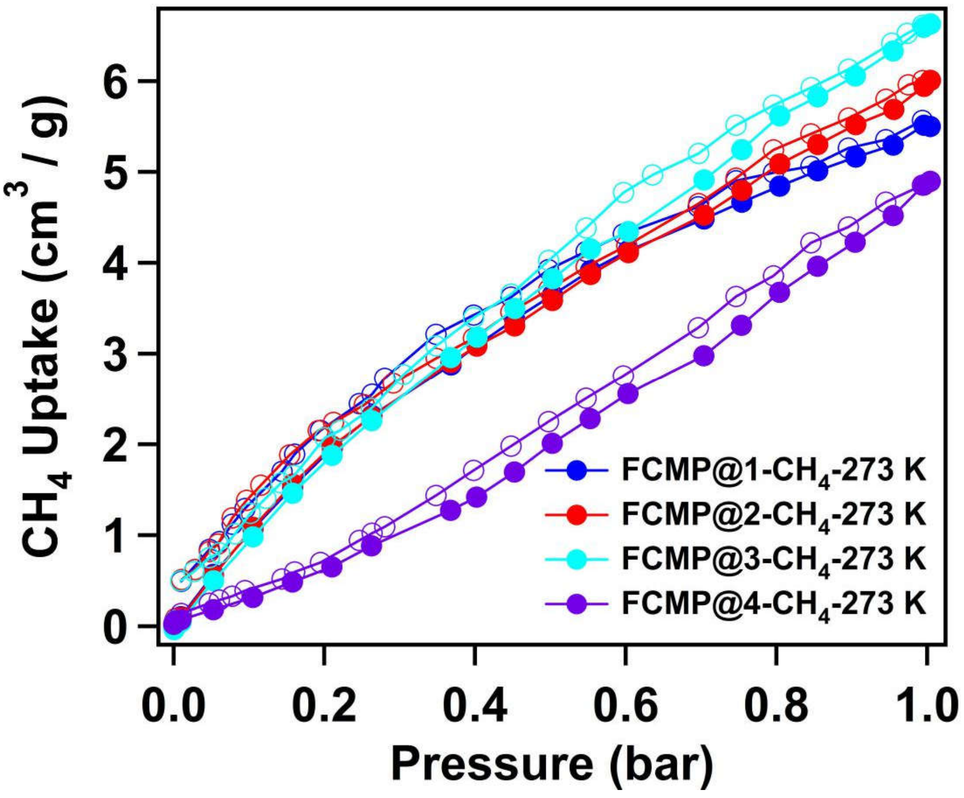

Figure S9 | CH_4 uptake of polymers FCMP@1-4 at 273 K and 1.0 bar.

SectionJ. Gas selectivity analyses

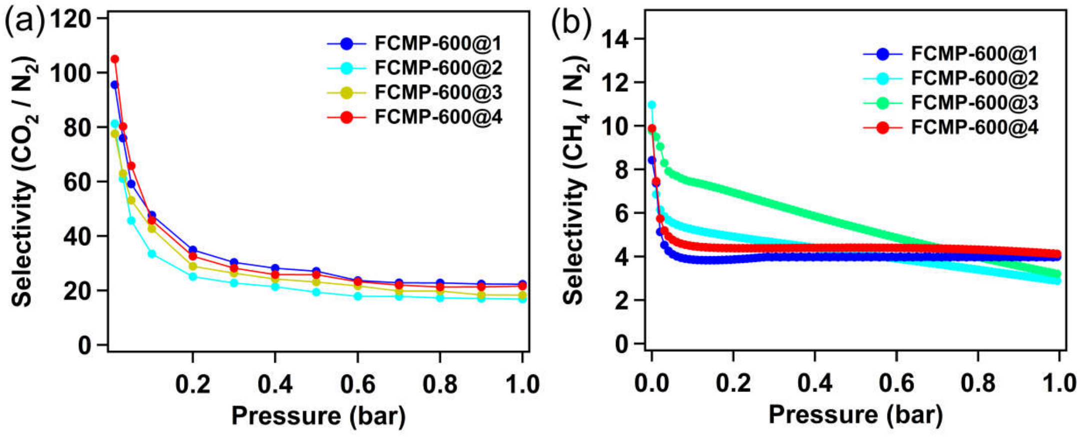

Figure S10 | (a) CO_2/N_2 selectivity of FCMP-600@1-4 for a molar ratio of 15/85 at 273 K; (b) CH_4/N_2 selectivity of FCMP-600@1-4 for a molar ratio of 50/50 at 273 K.

Section K. Iodine capture analyses

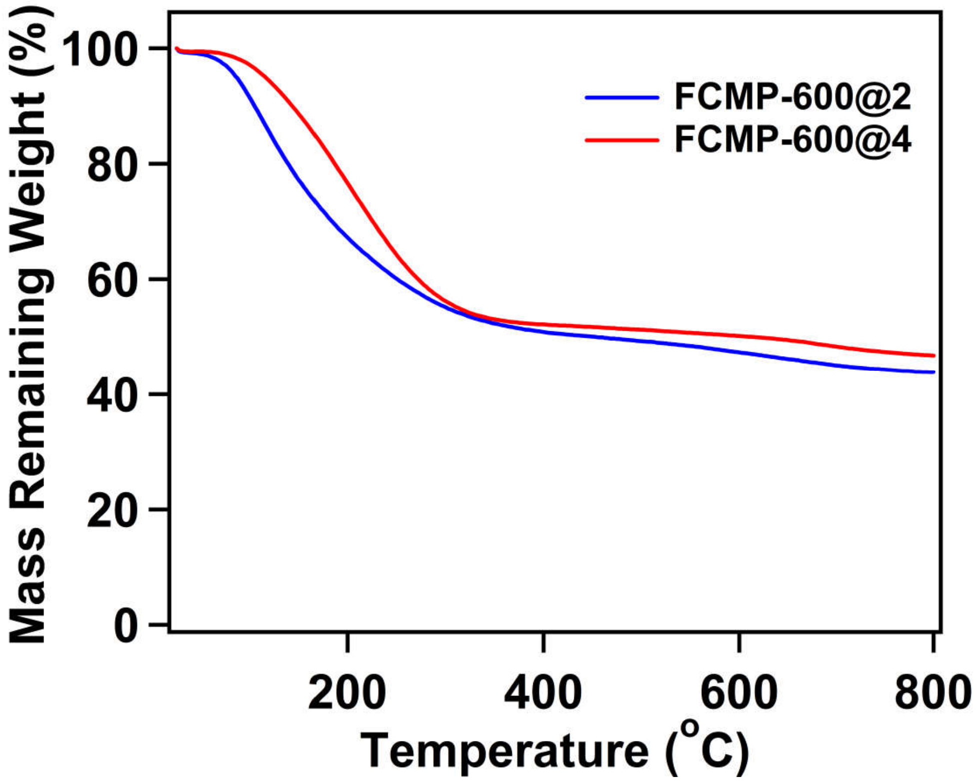

Figure S11 | TGA trace of I_2@FCMP-600@2 and I_2@FCMP-600@4.

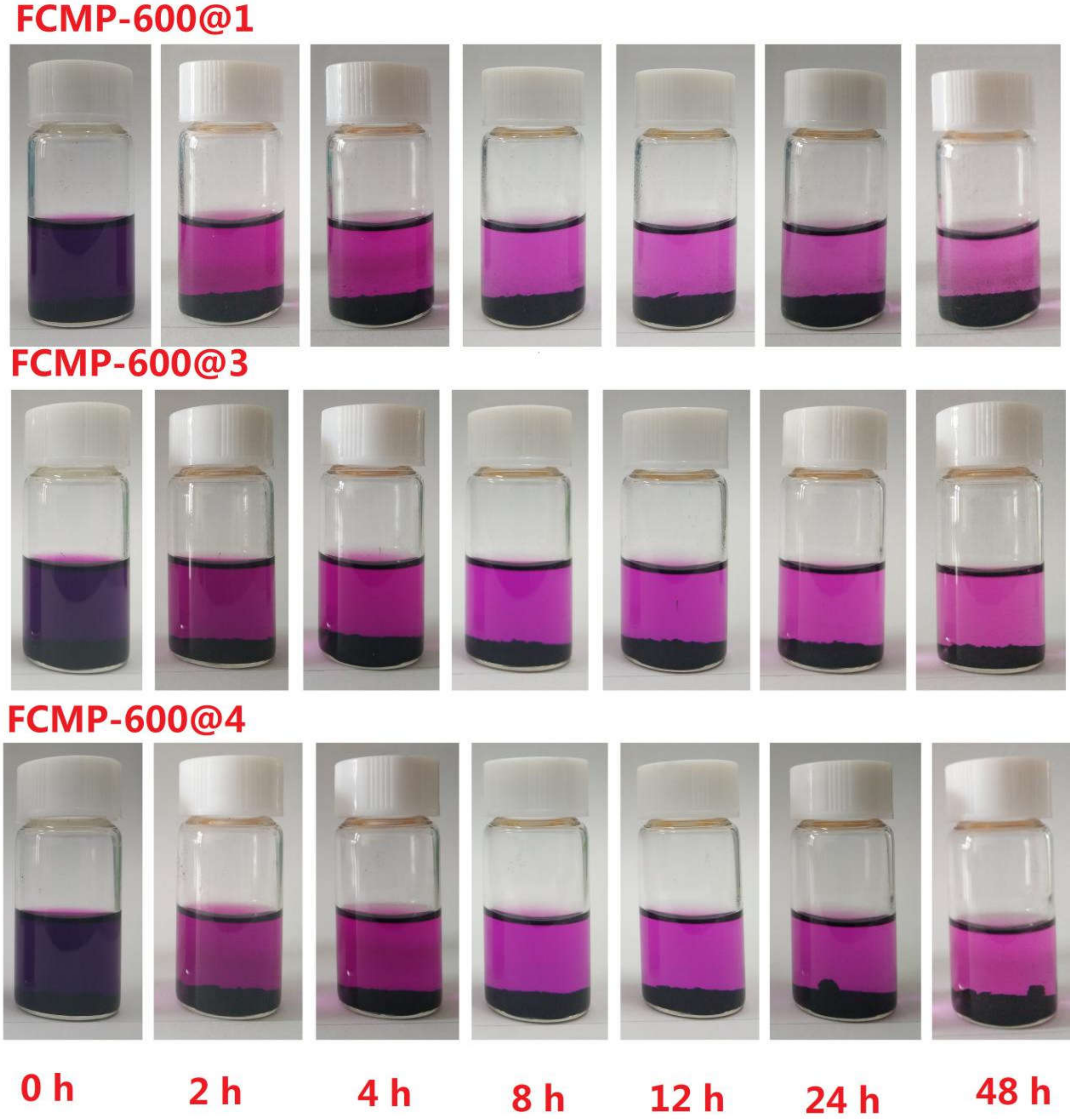

Figure S12 | The photographs show the different iodine adsorption rates of FCMP-600@1, FCMP-600@3, and FCMP-600@4.

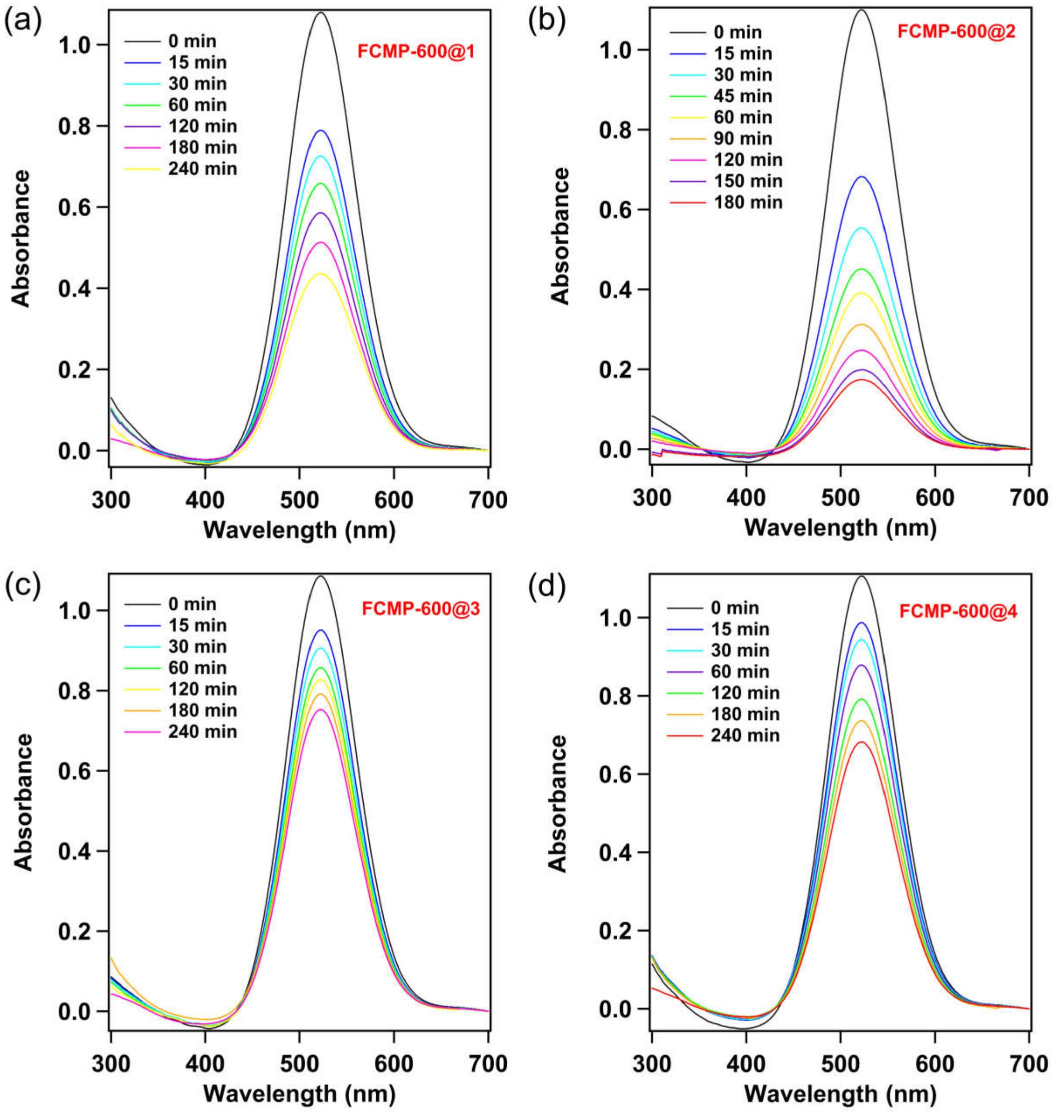

Figure S13 | UV/Vis spectra upon immersion of 30 mg FCMP-600@1-4 in hexane solution of I_2 (10^{-2} M). All experiments were performed at ambient temperature and pressure.

(1) Sorption Kinetic Studies

The linear form of the pseudo-first-order kinetic model:

$$\ln(Q_e - Q_t) = \ln Q_e - k_1 t$$

Where Q_t and Q_e are the mass percent of iodine adsorbed at time t and equilibrium (%), k_1 is the pseudo-first-order rate constant of adsorption process (h^{-1}).

The linear form of the pseudo-second-order kinetic model:

$$\frac{t}{Q_t} = \frac{1}{k_2 Q_e^2} + \frac{t}{Q_e}$$

Where Q_t and Q_e are the mass percent of iodine adsorbed at time t and equilibrium (%), k_2 is the pseudo-second-order rate constant of adsorption process ($(\%\ h)^{-1}$).

Table S2 | Parameters of the different isotherm models extracted from the pseudo-first-order model and the pseudo-second-order modelfor FCMP-600@1-4.

Adsorbent	Pseudo-first-order			Pseudo-second-order		
	k_1 (1/h)	Q_e (%)	R^2	k_2 (1/h)	Q_e (%)	R^2
**FCMP-600@1**	1.694	91.01	0.8778	0.0412	93.25	0.9913
**FCMP-600@2**	1.4795	89.24	0.8809	0.0318	92.13	0.9938
**FCMP-600@3**	1.682	89.09	0.8646	0.0412	91.39	0.9864
**FCMP-600@4**	1.0018	81.15	0.8768	0.0177	85.81	0.9937

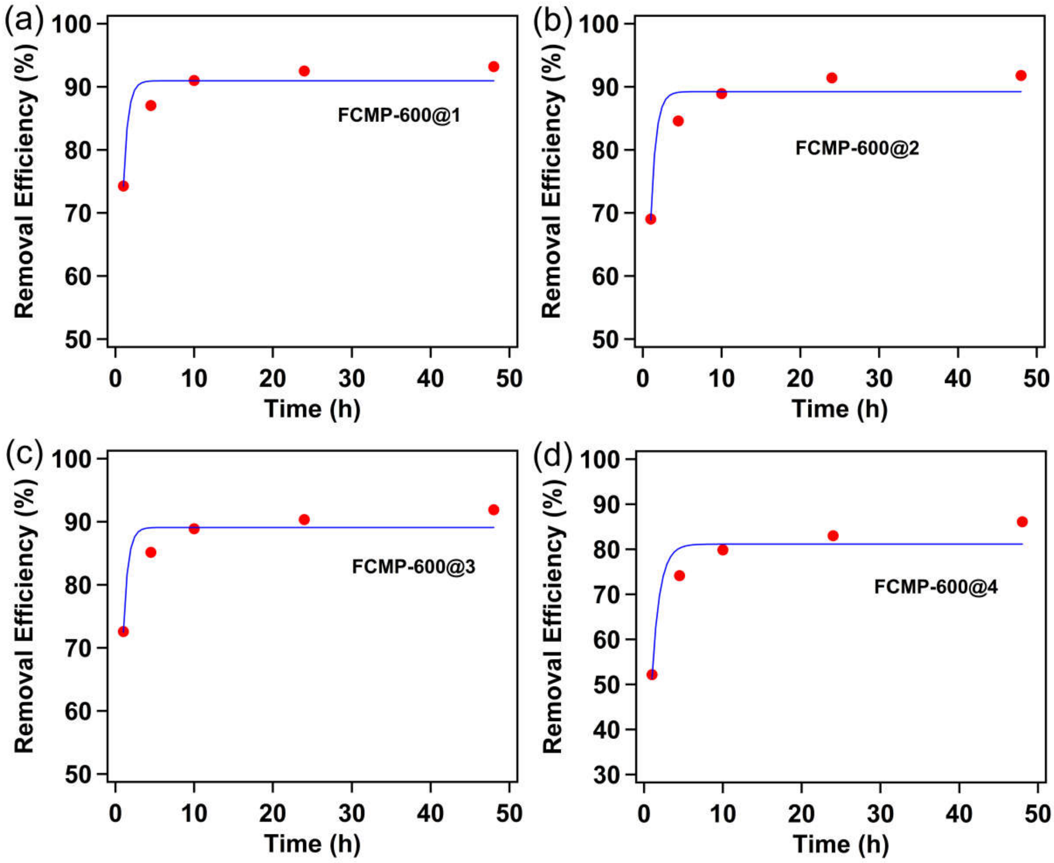

Figure S14 | The iodine adsorption kinetic was analyzed by the pseudo-first-order model. Initial concentration of iodine solution: 4 mg mL^{-1}.

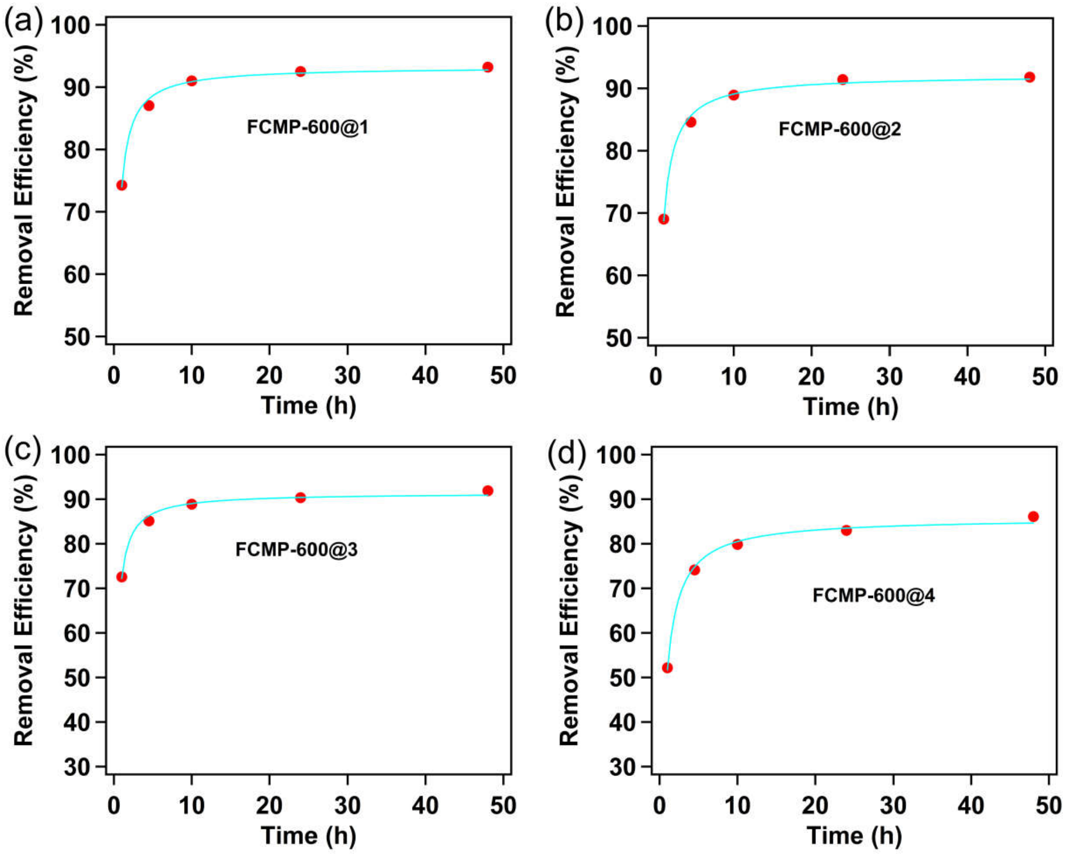

Figure S15 | The iodine adsorption kinetic was analyzed by the pseudo-second-order model. Initial concentration of iodine solution: 4 mg mL^{-1}.

(2) XPS Spectrum

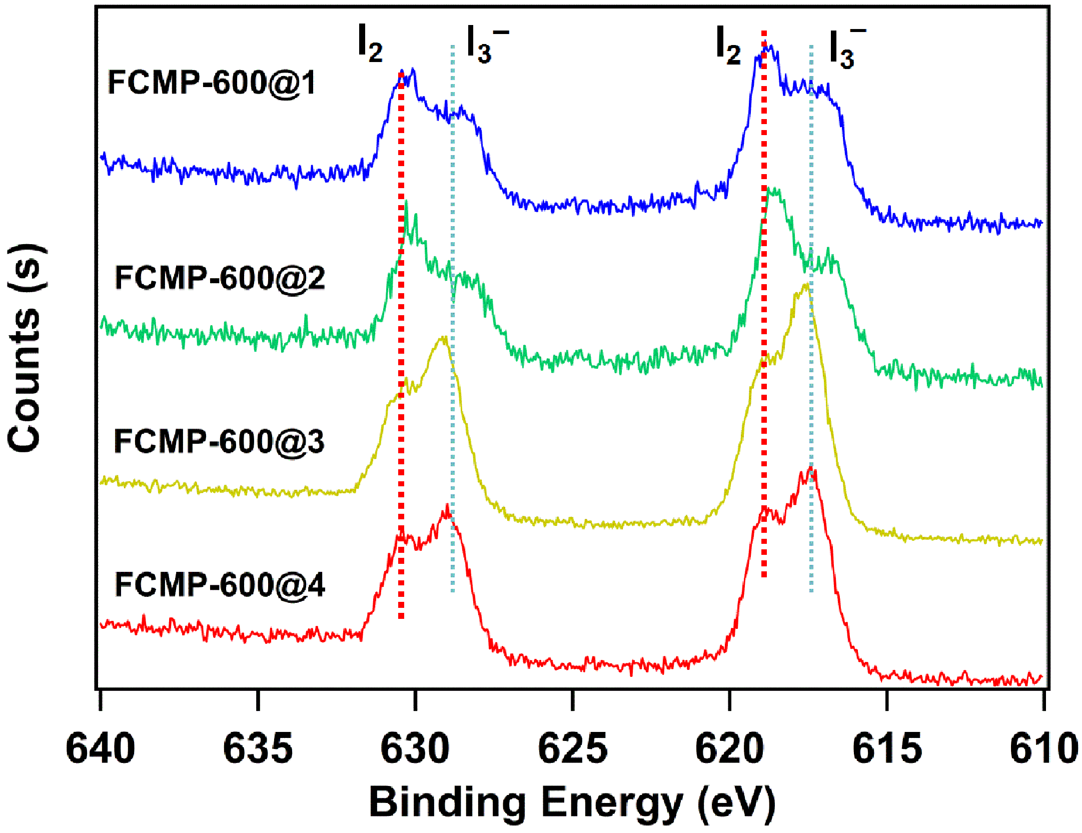

Figure S16 | XPS spectrum of FCMP-600@1-4 after iodine capture.

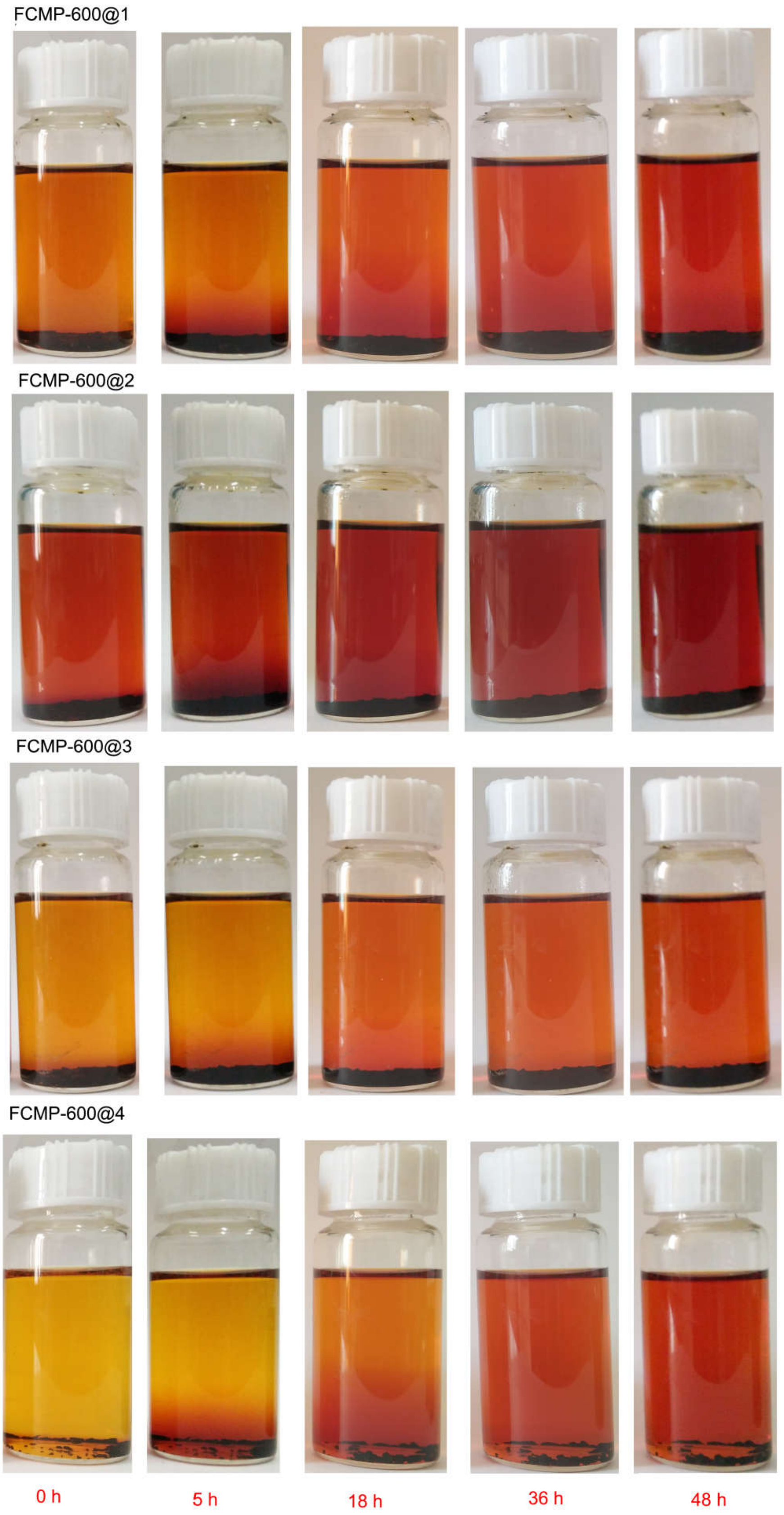

Figure S17 | Photos of FCMP-600@1-4 for iodine released.

(3) Recyclability for Iodine Uptake

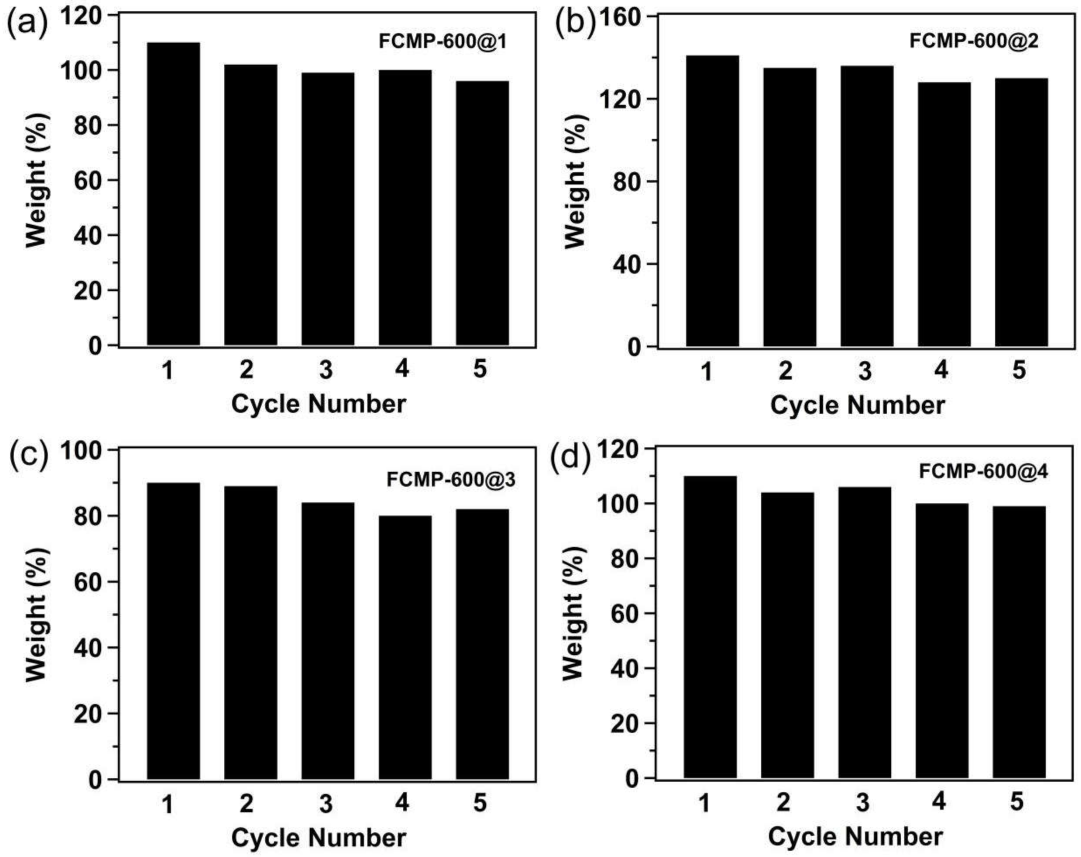

Figure S18 | Reusability of FCMP-600@1-4 for iodine adsorption by vapor sublimation.

(4) Adsorption Isotherm Models

The Langmuir isotherm model:

$$Q_e = \frac{Q_m k_L C_e}{1 + k_L C_e}$$

The Freündlich isotherm model:

$$Q_e = k_F C_e^{\frac{1}{n}}$$

Where k_L (mg^{-1}) and Q_m (mg g^{-1}) are the Langmuir isotherm constants; k_F (mg^{-1}) and nare the Freündlich isotherm constants; C_e is the concentration at equilibrium (mg mL^{-1}), Q_e is the amount of iodine adsorbed at equilibrium (mg g^{-1}).

Table S3 | Parameters of two simulation models extracted from experimental adsorption isotherms data for FCMP-600@1-4.

Adsorbent	Langmuir isotherm			Freundlich isotherm		
	Q_m (mg/g)	k_L(l/mg)	R^2	k_F (l/mg)	n	R^2
**FCMP-600@1**	1264	0.077	0.9455	123	1.284	0.9749
**FCMP-600@2**	2090	0.054	0.9852	128	1.62	0.9345
**FCMP-600@3**	960	0.1182	0.9335	180	1.4	0.9324
**FCMP-600@4**	1337	0.0677	0.9697	101	1.362	0.9615

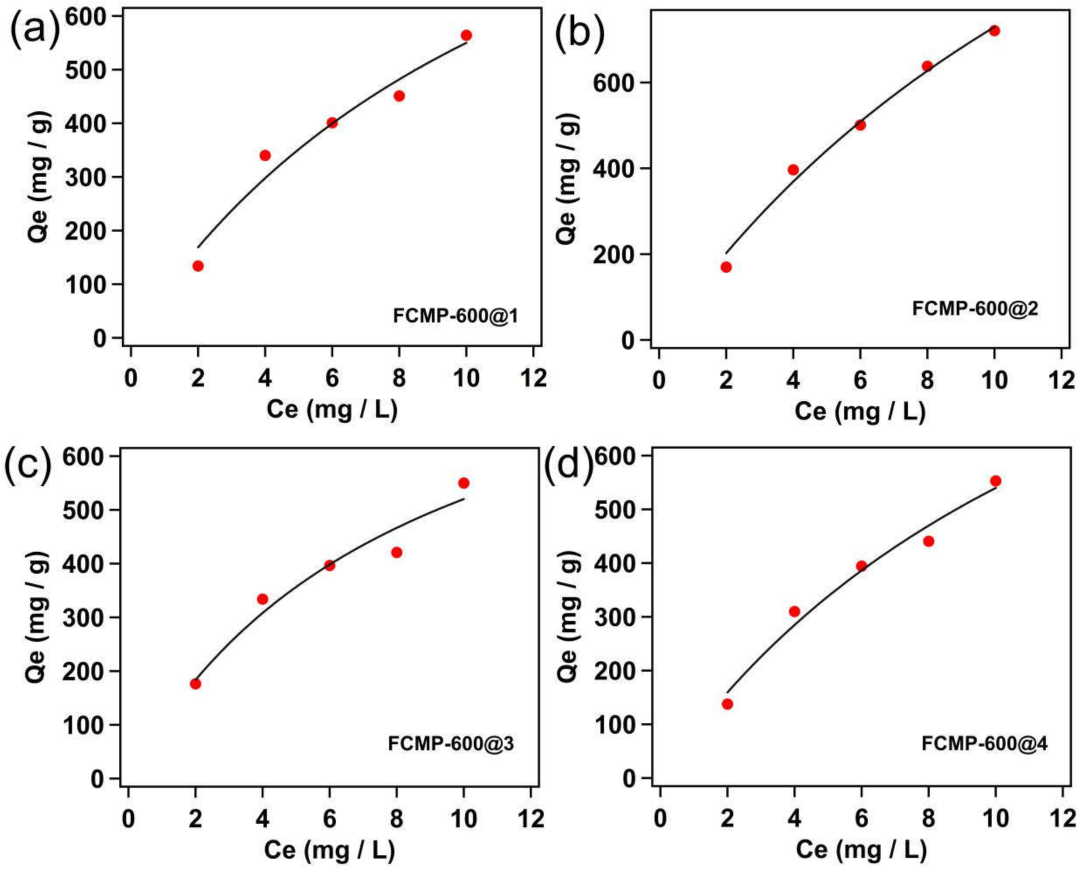

Figure S19 | Adsorption isotherm of FCMP-600@1-4 for iodine (30 mg polymer wassoaked in 3 mL of iodine solution at various concentrations for 48 h). Fitting curve: Langmuir.

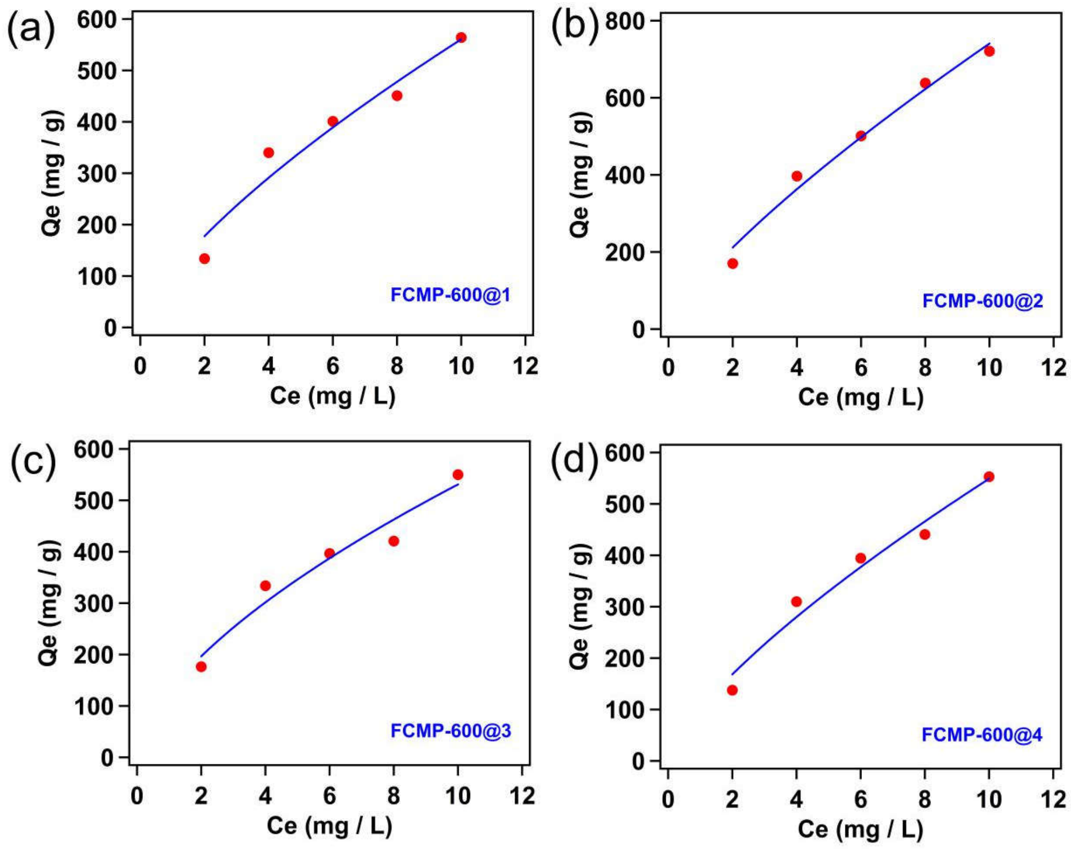

Figure S20 | Adsorption isotherm of FCMP-600@1-4 for iodine (30 mg polymer wassoaked in 3 mL of iodine solution at various concentrations for 48 h). Fitting curve: Freundlich.

Section L. Supporting references

S1.Li, P. Z., Wang, X. J., Liu, J., Lim, J. S., Zou, R. & Zhao, Y. A triazole-containing metal–organic framework as a highly effective and substrate size-dependent catalyst for CO_2conversion. *J. Am. Chem. Soc.* **138**, 2142-2145 (2016).

S2. Schultz, A., Laschat, S., Diele, S. & Nimtz, M. Tetraphenylethene-derived columnar liquid crystals and their oxidative photocyclization. *Eur. J. Org. Chem.* 2829-2839 (2003).

S3.Wang,J., Mei, J., Zhao, E., Song, Z., Qin, A., Sun, J. Z. & Tang, B. Z. Ethynyl-capped hyperbranched conjugated polytriazole: click polymerization, clickable modification, and aggregation-enhanced emission. *Macromolecules* **45**, 7692-7703 (2012).
